# Supplementary material for: Spatiotemporal formation of glands in plants is modulated by MYB-like transcription factors
Source: Nat Commun. 2024 Mar 15;15:2303. doi: 10.1038/s41467-024-46683-0 (PMC10943084; doi:10.1038/s41467-024-46683-0)
Supplement: Supplementary file 1 — Supplemantary information [file 41467_2024_46683_MOESM1_ESM.pdf]

Spatiotemporal formation of glands in plants is modulated by MYB-like transcription factors

Jiang Chang[1, 2], Shurong Wu[1, 2], Ting You[1, 2], Jianfeng Wang[1], Bingjing Sun[1], Bojun Xu[1], Xiaochun Xu[1], Yaping Zhang[1], Shuang Wu[1]*

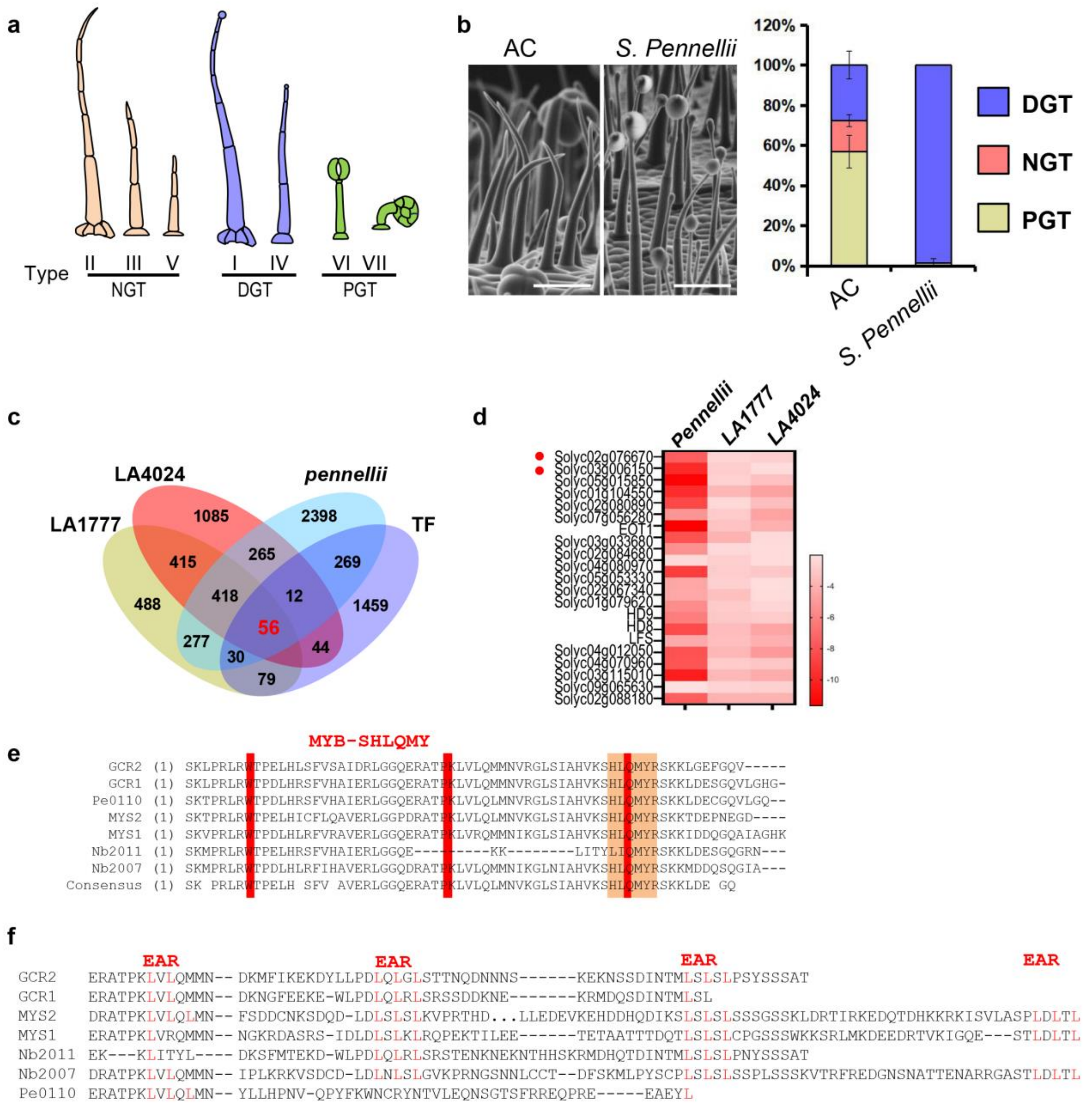

Supplementary Figure 1. Screening for key genes regulating gland development.

a Seven types of trichomes in tomato (type I-VII). Based on the morphology and presence of glandular cell, seven types of trichomes are divided into three categories: NGT (non-glandular trichomes including type II, III and V), DGT (digital glandular trichomes including type I and IV) and PGT (peltate glandular trichomes including type VI and VII). **b** SEM observation and qualification of trichomes on the *S. Pennellii* LA0716. The Y-axis represents the proportion of the three categories of trichomes in the total number of trichomes. Bar: 200 μm. *p*-values were calculated by unpaired two-sided *t*-test and exact *p*-values are presented in the Supplementary Table 8. **c** Venn diagram showing the overlap of up-regulated transcription factors in glandular trichomes of *S. Pennellii* LA0716, *S. habrochaites* LA1777 and tomato cultivar *S. lycopersicum* LA4024 (log2FoldChange (leaf/glandular trichome) ≤ -1). Transcriptome data of LA4024 and LA1777 was cited from Balcke et al (2016) (25). Transcription factors in tomato were obtained from iTAK (http://itak.feilab.net/cgi-bin/itak/db_browse.cgi). **d** Heat-map showing transcription

factors highly expressed in the glandular trichomes of *S. Pennellii* LA0716, *S. habrochaites* LA1777 and *S. lycopersicum* LA4024 based on the transcriptome data. Transcription factors with log2FoldChange (leaf/glandular trichome) ≤ -2 are shown. Red dots indicate two transcription factors with 81% homology. **e** The MYB-like domain with SHLQMY motif in the N-terminal of GCR1/2. **f** The EAR motif in the C-terminal of GCR1/2 and its homologs.

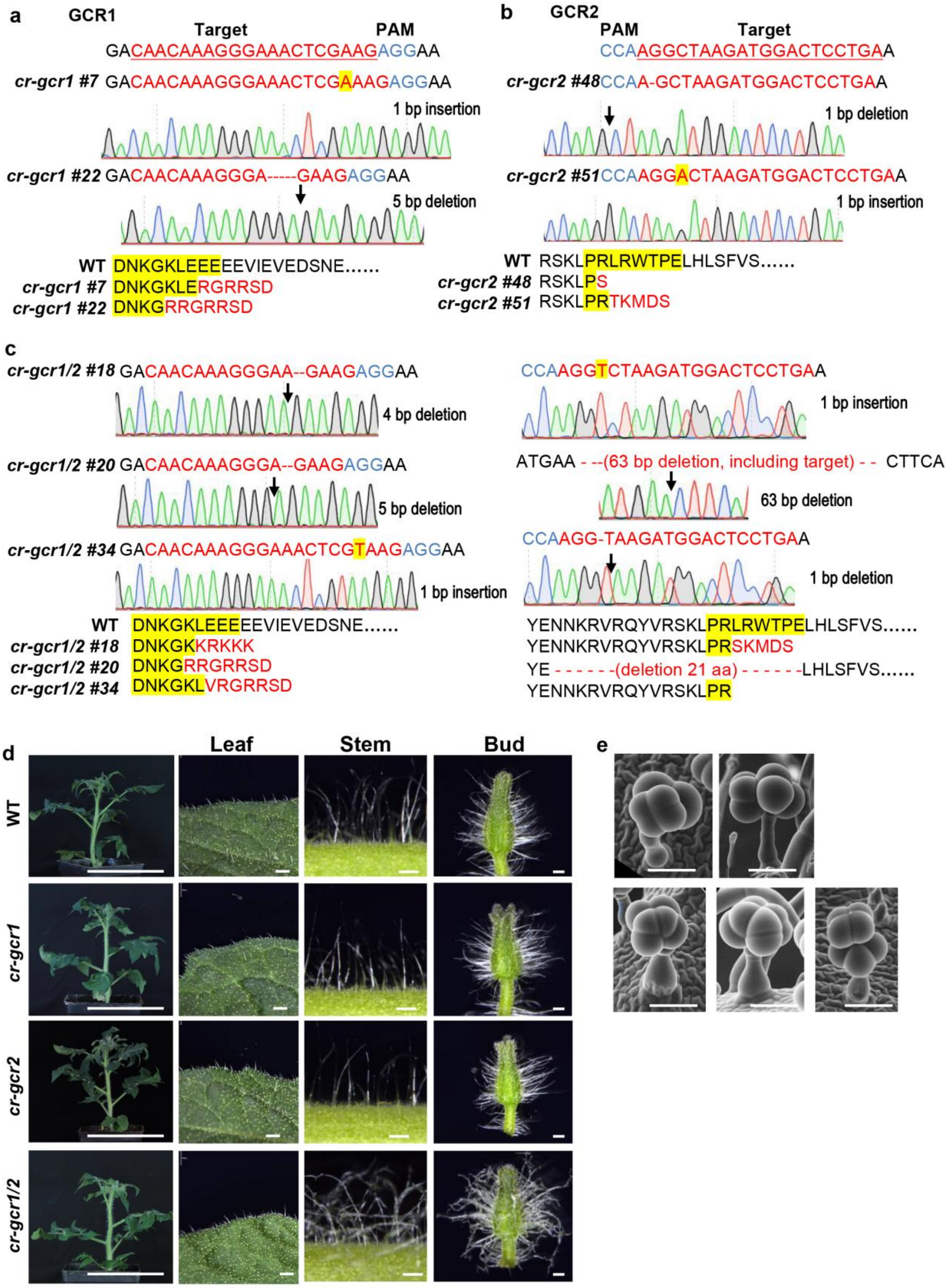

Supplementary Figure 2. Knockout of GCR1/2 promotes gland formation in micro-tom tomato.

a-**c** Sequence analysis of *gcr1/2* single (*cr-gcr1, cr-gcr2*) and double (*cr-gcr1/2*) mutants generated by CRISPR/Cas9. Both single and double mutants were obtained by stable transformation. Sequences with red underlines are the single guide RNA target sequences of *GCR1* and *GCR2*, which are the same in the single and double mutants. Sequences in blue (AGG, CCA) are the protospacer-adjacent motif (PAM)

sequences. There are 1 base-pair insertion and 5 base-pair deletions of *GCR1* in lines 7 and 22 of *gcr1* single mutant, all resulting in the truncated proteins (highlighted in yellow) **(a)**. There are 1 base-pair deletion and 1 base-pair insertion of *GCR2* in lines 48 and 51 of *gcr2* single mutant, all resulting in the truncated proteins (highlighted in yellow) **(b)**. There are 4 base-pair deletions, 5 base-pair deletions and 1 base-pair insertion of *GCR1* in lines 18, 20 and 34 of *gcr1/2* double mutant, all resulting in the truncated proteins (highlighted in yellow) . There is a 1 base-pair deletion of *GCR2* in lines 18 and 34 of *gcr1/2* double mutant, resulting in the truncated proteins and a 63 base-pair deletion in line 20 which results in a 21 amino acid deletion (highlighted in yellow)**(c)**. **d** The plants, leaves, stems and buds of *gcr1* mutant (*cr-gcr1*), *gcr2* mutant (*cr-gcr2*) and *gcr1/2* double mutant (*cr-gcr1/2*). Bar: 10 cm (plants), 1 mm (Leaves, stems and buds). **e** PGT of *gcr1/2* double mutants (*cr-gcr1/2*) has more than one giant gland with distinct morphology. Bar: 50 μm.

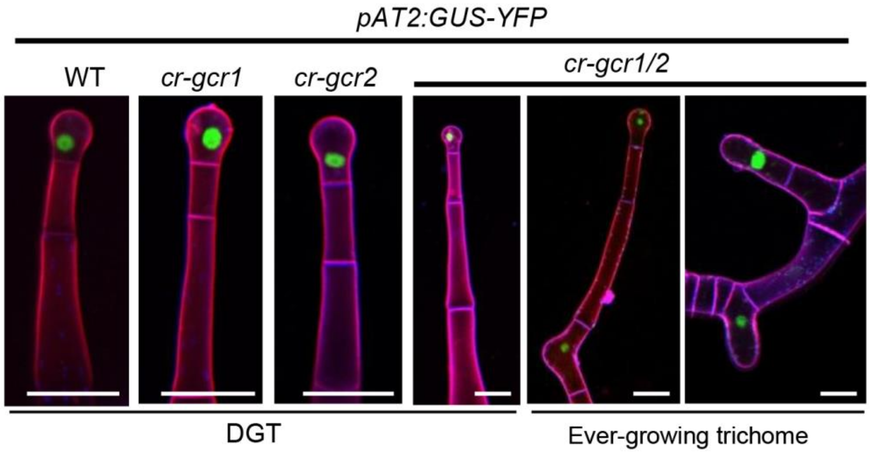

Supplementary Figure 3. Visulization of glandular marker lines.

Marker of glandular cells (*pAT2*:*GUS*-*YFP*) is introduced into *gcr1* and *gcr2* single mutants and *gcr1/2* double mutant (*cr*-*gcr1/2*). Propidium Iodide staining is used to show the cell edge. Chloroplast auto-fluorescence is shown in blue. YFP is shown in green. All images processed by maximum intensity projection of z-stacks. Bar: 40 µm.

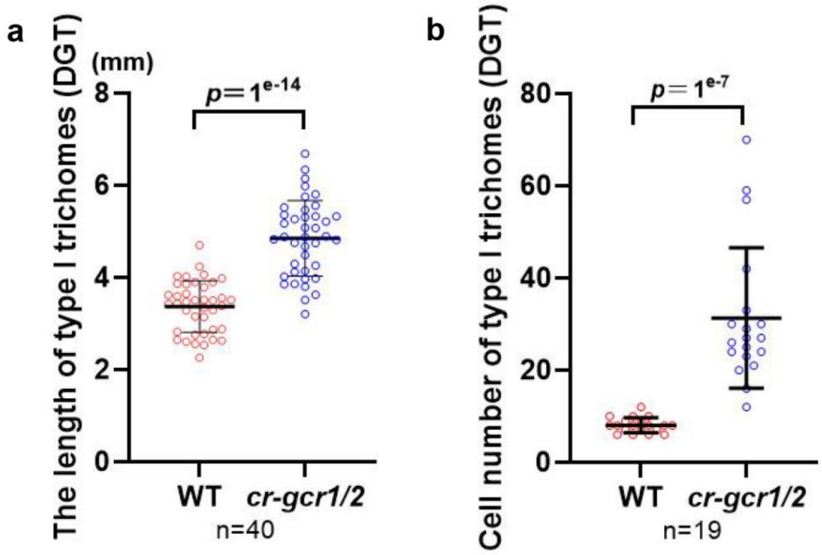

Supplementary Figure 4. Quantification of trichome length a and cell number b of *cr-gcr1/2*. n represents the number of trichomes used for the quantification. At least 19 trichomes from two lines were used to quantify. Data are shown as mean ± SD. *p*-values were calculated by unpaired two-sided *t*-test.

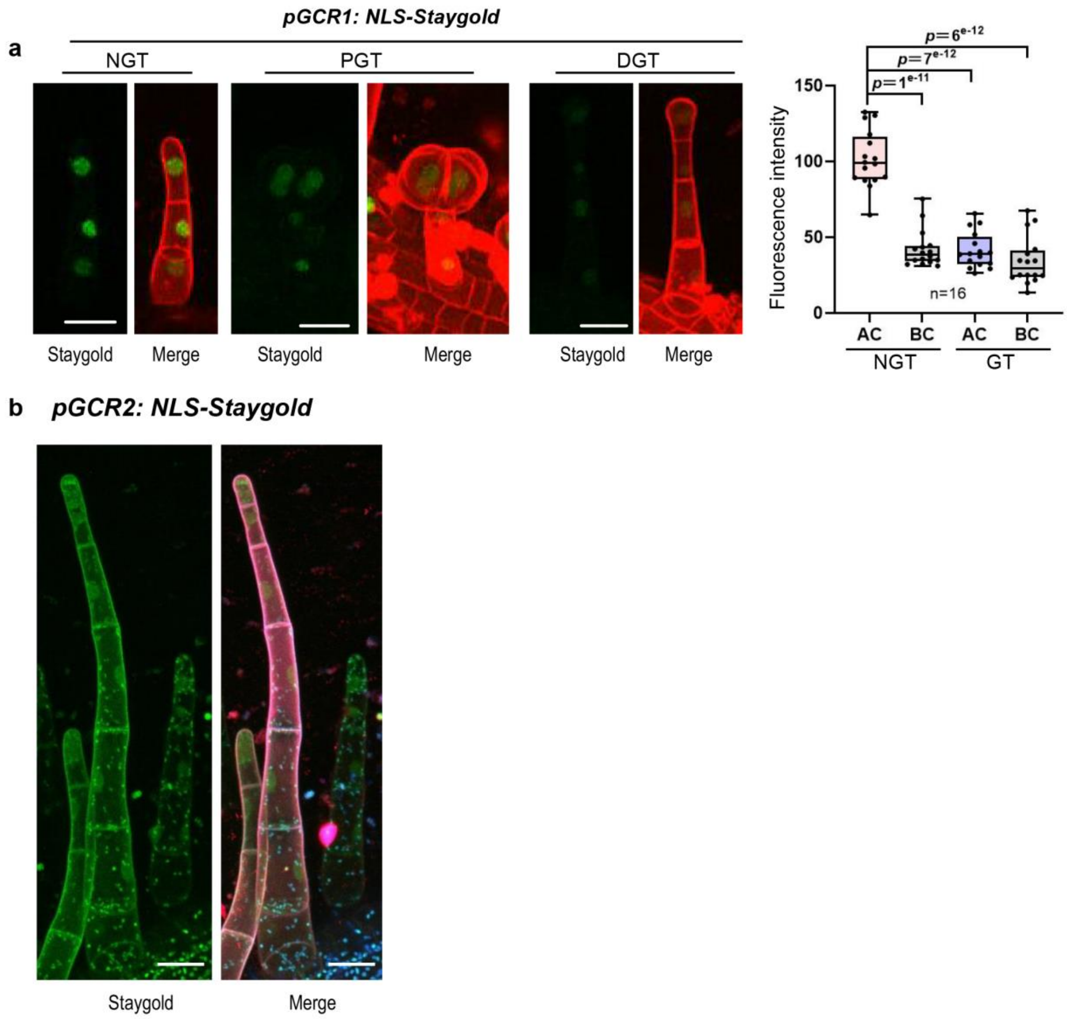

Supplementary Figure 5. Expression pattern of *GCR1* (a) and *GCR2* (b). Propidium Iodide staining is used to show the cell edge. Stadygold is shown in green. BC: Basal cell; AC: Apical cell. GT: glandular trichomes including DGT and PGT. Bar: 50 µm **(a)**, 20 µm **(b)**. GCR1 expression is quantified by fluorescence intensity measurement of z-stacked images of *pGCR1:NLS-Staygold*. Box plots show maximum, minimum, first and third quartiles, median (line). n represents the number of trichomes used for the quantification. 16 trichomes from two lines were used to quantify. *p*-values obtained by unpaired *t*-test.

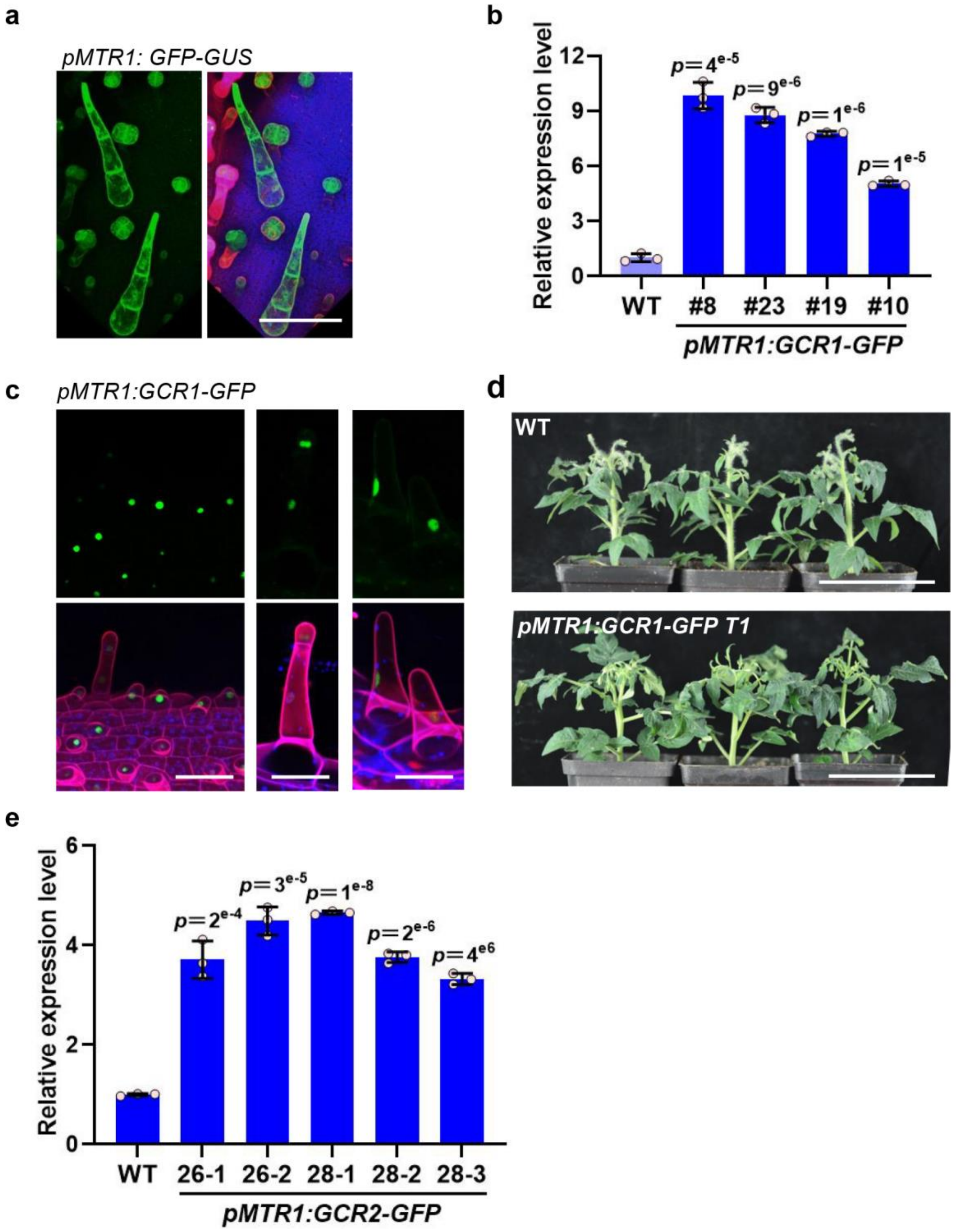

Supplementary Figure 6. Expression of *GCR1* and *GCR2* inhibits gland formation.

a Expression pattern of *MTR1*. Auto-fluorescence of chloroplast is shown in blue. Fluorescence of PI is shown in red. **b** Relative expression level of *GCR1* in the *pMTR1:GCR1-GFP* transgenic plants. Data are shown as mean ± SD (n=3 biological replicates). *p*-values obtained by unpaired *t*-test. **c** GCR1-GFP protein in *pMTR1:GCR1-GFP* plants. GCR1-GFP protein in nuclei is shown in green. Fluorescence of PI is shown in red. Bar: 20 μm. **d** Transgenic plants expressing *GCR1* driven by *MTR1* promoter. Bar: 10 cm. **e** Expression level of *GCR2* in *pMTR1:GCR2-GFP*. Data are shown as mean ± SD (n=3 biological replicates). *p*-values obtained by unpaired *t*-test.

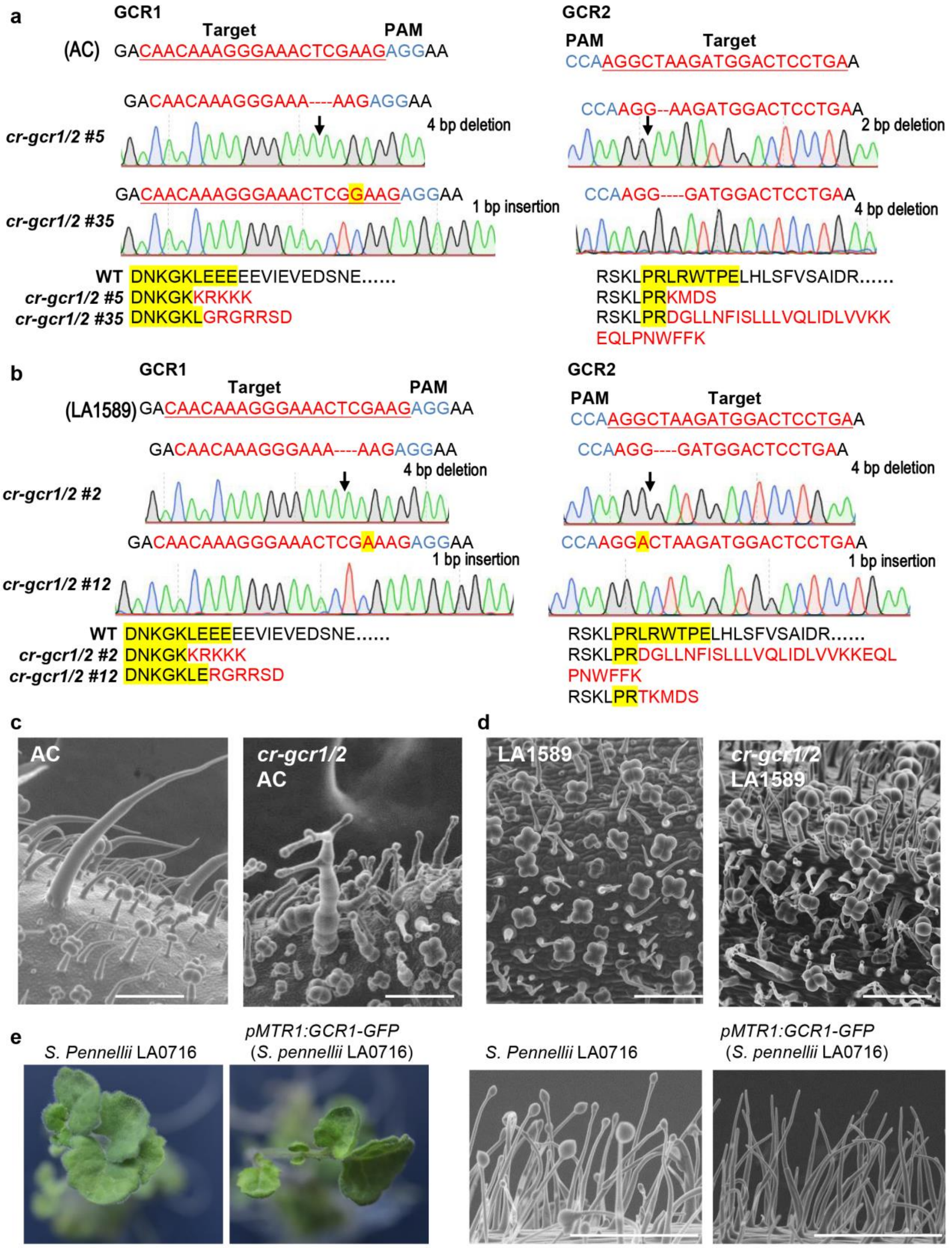

Supplementary Figure 7. Verification of GCR1/2 function in Alisa Craig (AC), *S. pimpine* LA1589 and *S.pennellii* LA0716.

a and b Sequence analysis of *gcr1/2* double mutants (*cr-gcr1/2*) generated by CRISPR/Cas9 in AC **(a)** and LA1589 **(b)**. Sequences with red underlines are the single guide RNA target sequences of GCR1 and GCR2. **c and d** SEM images show that knockout of *GCR1/2* in AC and LA1589 promotes gland formation. Bar: 100 μm. **e** Expression of *GCR1* driven by *MTR1* promoter inhibits gland formation in *S.pennellii.* Bar: 300 μm (SEM images). At least thirty lines were obtained.

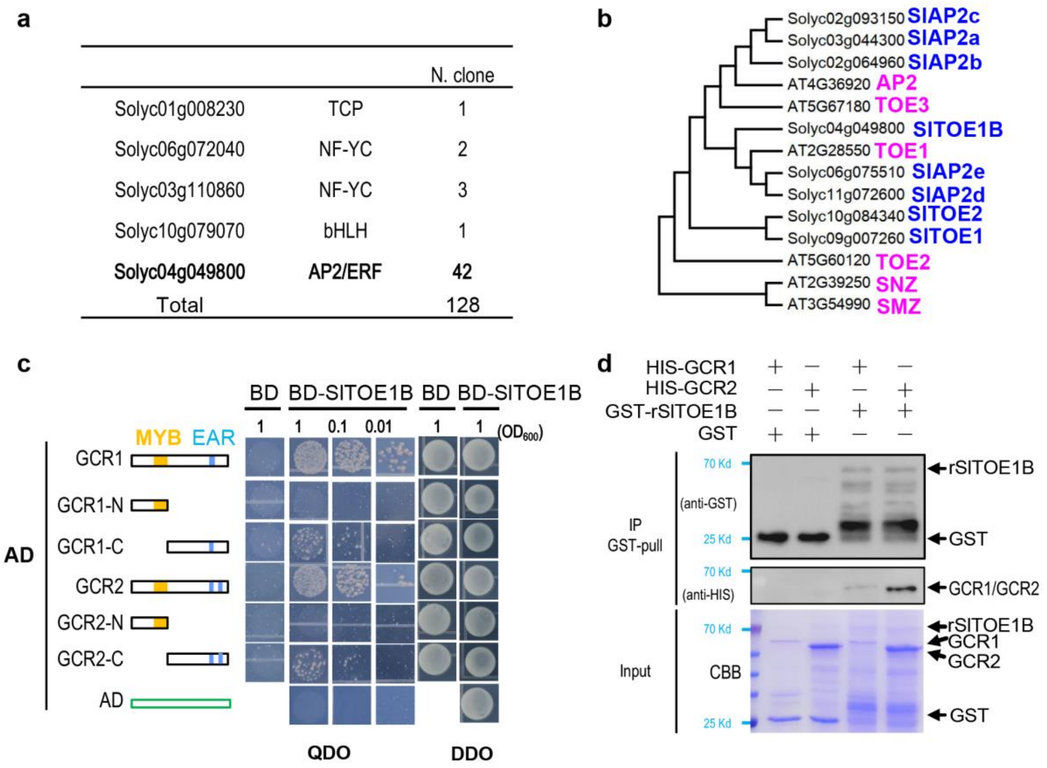

Supplementary Figure 8. GCR1/2 interact with SlTOE1B.

a Yeast-two-hybrid (Y2H) cDNA library screening for GCR1-interacting factors. A total of 128 clones are sequenced, and 42 are the AP2/ERF transcriptional factor Solyc04g049800. **b** Phylogenic analysis of TOE homology in tomato and *Arabidopsis*. Protein sequences of TOE homology are used. **c** Y2H assays show GCR1/2 interact with SlTOE1B through its C-terminal domain. N-terminal domain of GCR1 (GCR1-N), C-terminal domain of GCR1 (GCR1-C), GCR2-N and GCR2-C are inserted into pGADT7 vector with DNA Gal4-activation domain (AD). SlTOE1B is cloned into pGBKT7 vector with DNA-binding domain (BD). The black boxes represent the coding sequence of GCR1, GCR2 and their fragments used for the interaction verification. The yellow boxes represent the MYB-like domain of GCR1 and GCR2. The blue boxes represent the EAR motif of GCR1 and GCR2. Interaction is tested with gradient cell dilutions (1, 0.1 and 0.01 OD_{600}). DDO: SD/-Leu-Trp. QDO: SD/-Ade-His-Leu-Trp. Data represent results of three independent experiments. **d** GST-pull down assay show that GST-SlTOE1B but not GST pulls down HIS-GCR1 and HIS-GCR2. GST beads are used for co-immunoprecipitation and His antibody is used to detect the GCR proteins. Data represent results of three independent experiments.

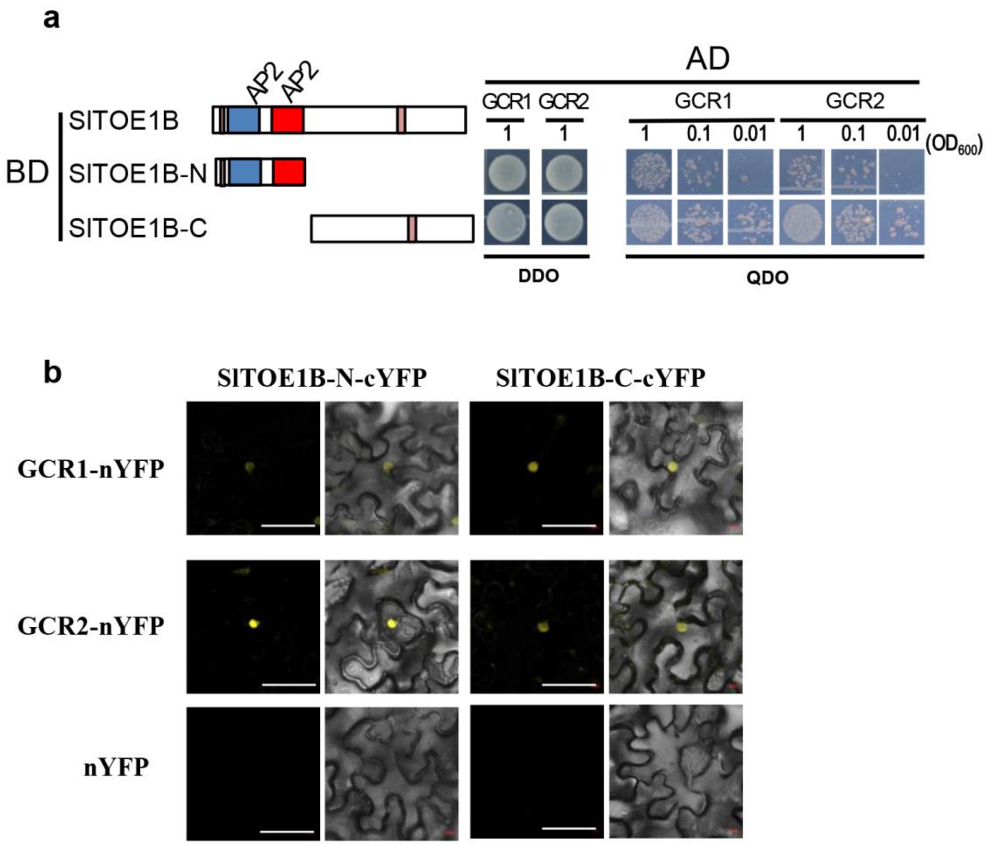

Supplementary Figure 9. N-terminal and C-terminal domains of SlTOE1B interact with GCR1/2.

a Y2H assay. N-terminal and C-terminal domains of SlTOE1B are fused with DNA binding domain (BD). GCR1/2 are fused with DNA activation domain (AD). **b** BiFC assays in *N. benthamiana* leaves. N-terminal and C-terminal domains of SlTOE1B are fused with cYFP. GCR1/2 are fused with nYFP. Bar: 50 μm. Data represent results of three independent experiments in a and b.

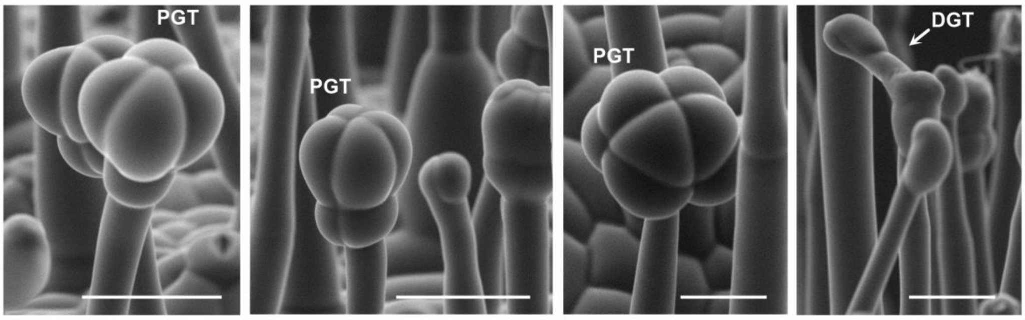

Supplementary Figure 10. **PGT and DGT of *pMTR1:SlTOE1B* plants form more than one glanular cells**. Bar: 50 μm.

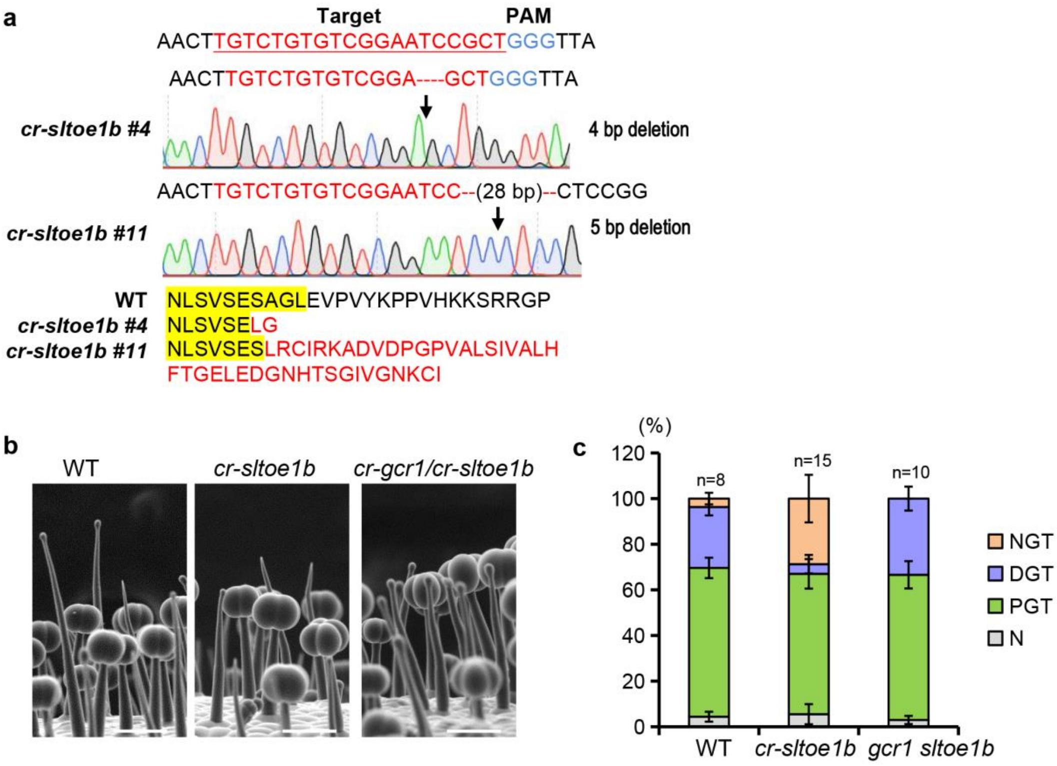

Supplementary Figure 11. **Sequence analysis of *sltoe1b* mutant and phenotype of *sltoe1b gcr1* double mutant.**

a Sequence analysis of *sltoe1b* mutant generated by CRISPR/Cas9. **b** Phenotypes of *cr-sltoe1b* and *sltoe1b gcr1* double mutant (*cr-sltoe1b/cr-gcr1*). **c** Quantification of the trichomes on the juvenile stems. The Y-axis represents the proportion of the three categories of trichomes in the total number of trichomes. n represents the number of SEM images used for the quantification. For each line, at least 8 different SEM images from four to five individual plants are used for trichome quantification. Data are shown as mean ± SD. *p*-values were obtained by unpaired two-sided *t*-test and exact *p*-values are presented in the Supplementary Table 9.

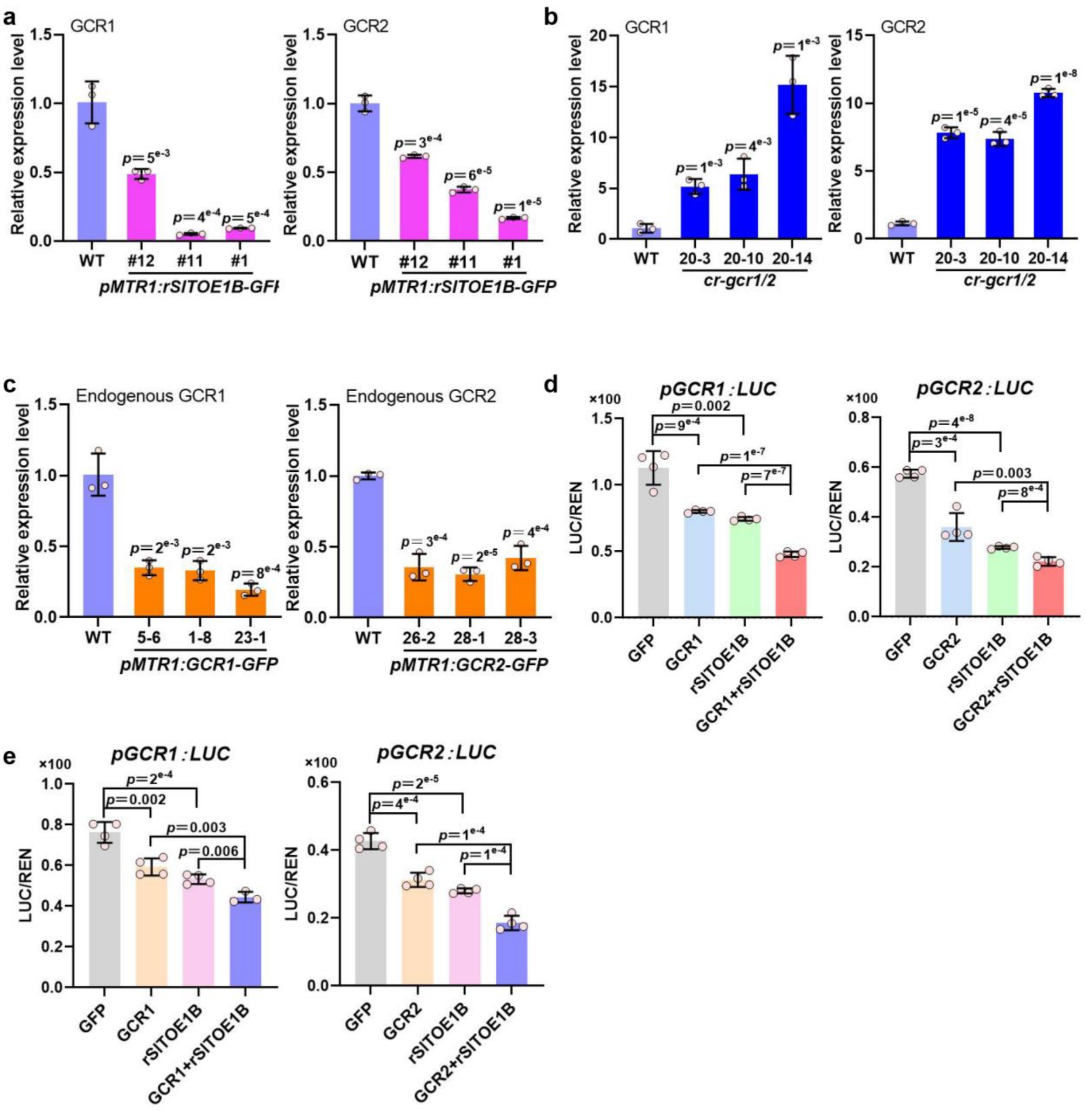

Supplementary Figure 12. *SlTOE1B* and *GCR1/2* regulate the expression of *GCR1/2*.

a Relative expression of *GCR1/2* in the *pMTR1:rSlTOE1B* transgenic plants. Data are shown as mean ± SD (n=3 biological replicates). *p*-values obtained by unpaired *t*-test. **b** Relative expression of *GCR1/2* in *gcr1/2* double mutants (*cr-gcr1/2*). Data are shown as mean ± SD (n=3 biological replicates). *p*-values obtained by unpaired *t*-test. **c** Relative expression of endogenous *GCR1* and *GCR2* in the *pMTR1:GCR1* and the *pMTR1:GCR2* transgenic plants. Data are shown as mean ± SD (n=3 biological replicates). *p*-values obtained by unpaired *t*-test. **d and e** LUC assay in WT **(d)** and *cr-gcr1/2* **(e)** protoplasts. GCR1/2 inhibits the transcriptional activity of *GCR1/2* promoters and such inhibition is significantly enhanced when SlTOE1B proteins are present. *p*-values were calculated by unpaired two-sided *t*-test. n = 3~4 biological replicates.

Motif	Sequence	*pGCR1*	*pGCR2*	*pLFS*	PJG-GCR1	PJG-GCR2	PJG	Promoter fragment
Motif1	AAGAATAA	-2837 ~ -2830<br>-808 ~ -801		-2173 ~ -2166(-)<br>-1330 ~ -1323(-)				*pGCR1-1; pLFS-2*
Motif2	AAGAATTA	-2097 ~ -2090	-2507 ~ -2500					
Motif3	GGGAATCT	-969 ~ -962						*pGCR1-2*
Motif4	GGGAATAA		-200~-193(-)					
Motif5	ACATTCTT			-2786 ~ -2779(-)				
Motif6	AAATTCTA	-2882 ~ -2875 (-)<br>-614 ~ -607		-1290 ~ -1283(-)<br>-246 ~ -239				*pLFS-4*
Motif7	AAATTCTT	-1174 ~ -1167 (-)		-163 ~ -156				*pLFS-5*
Motif8	ACATTCTC		-1890~ -1833	-92 ~ -85				*pLFS-5*
Motif9	ATATTCTT	-339 ~ -332 (-)	-2894 ~ -2887 (-)<br>-1423 ~ -1416 (-)	-30 ~ -23				*pLFS-5; pGCR2-1*
Motif10	TTATTCTC	-1581 ~ -1574 (-)	-2412 ~ -2405					*pGCR2-2*
Motif11	AGGAATAT	-1345 ~ -1338						
Motif12	GAGAATTT	-2508 ~ -2501 (-)						
Motif13	TAGAATTA	-2969 ~ -2962						
Motif14	TGGAATTA	-1711 ~ -1704(-)	-2984 ~ -2977<br>-2774 ~ -2767 (-)<br>-1379 ~ -1372 (-)					
Motif15	TGGAATAA		-2659 ~ -2652	-2257 ~ -2250(-)				
Motif16	AGGAATAA			-1067 ~ -1060(-)				
Motif17	AGGAATGA			-1543 ~ -1536(-)				
Motif18	GGGAATAT			-1731 ~ -1724(-)				

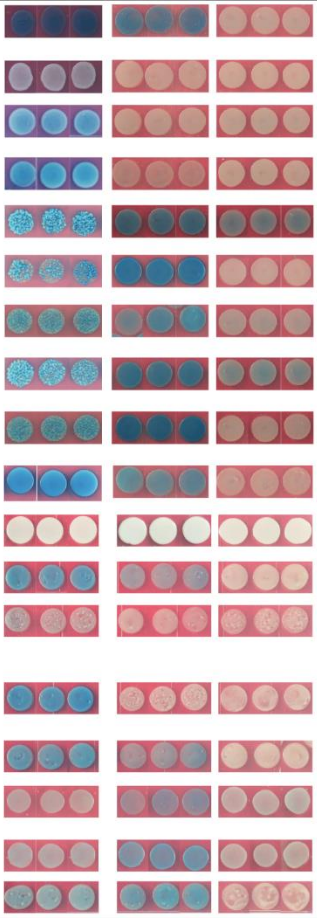

Supplementary Figure 13. Screening for GCR-binding motifs. Ten motifs are selected for the interaction verification. Data in columns 3-5 show the position of these motifs in *GCR1*, *GCR2* and *LFS* promoters. The last column shows the promoter fragments that contain the corresponding motifs and are used for Y1H assays (in support of Figure 6a and Figure 7d). Data represent results of three independent experiments.

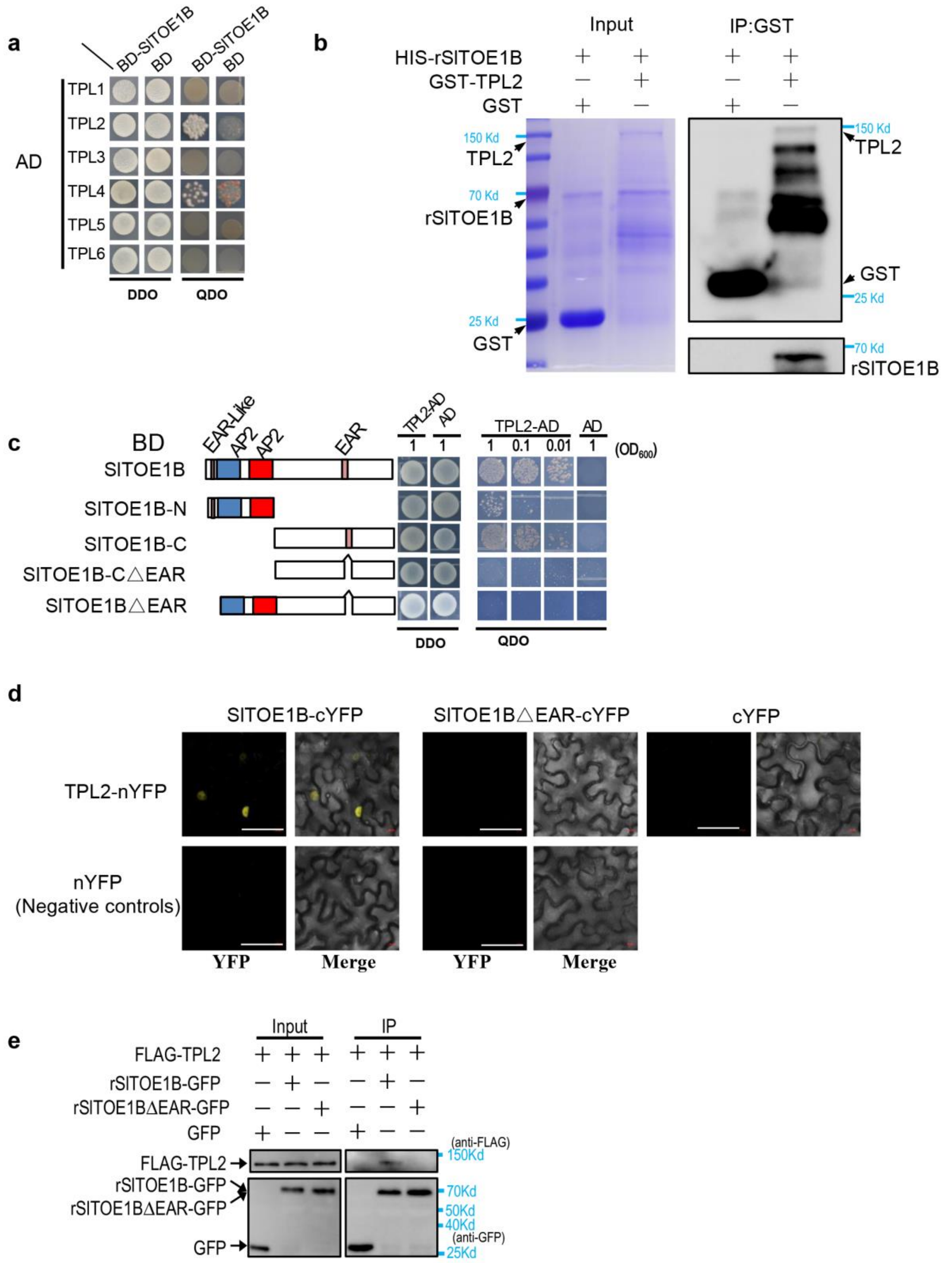

Supplementary Figure 14. SlTOE1B interacts with TPL2.

a Y2H assay. SlTPLs are in pGADT7 and SlTOE1B is in pGBKT7. DDO: SD/-Leu-Trp. QDO: SD/-Ade-His-Leu-Trp. **b** GST-pull down assay. GST-TPL2 but not GST pulls down His-SlTOE1B. GST beads are used for co-immunoprecipitation and His antibody is used to detect SlTOE1B protein. **c** Y2H shows that both EAR-like motif and EAR motif are required for the interaction between SlTOE1B and TPL2. **d** BiFC assays show that deletion of EAR and EAR-like motifs of SlTOE1B abolishes the interaction between SlTOE1B and TPL2. Bar: 50 μm. **e** Co-IP assay shows that

SlTOE1B interacts with TPL2. GFP (left lane; negative control) or GFP fused with the target protein (the other lanes) are used as bait to bind to the GFP beads. Immuno-precipitated proteins are detected by anti-FLAG antibody. Data represent results of three independent experiments including Y2H assays; GST-pull down assay; BiFC assays and Co-IP assay.

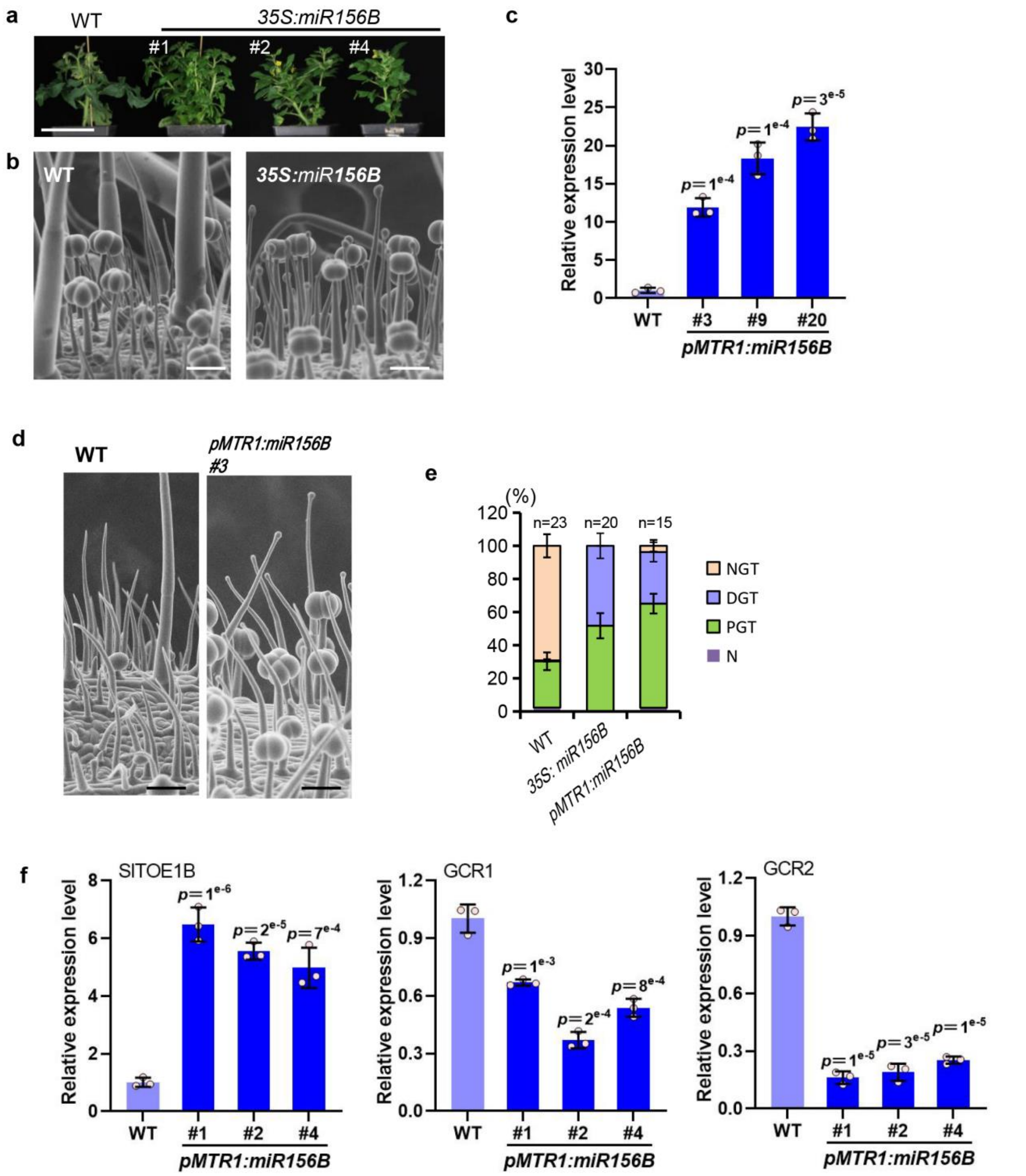

Supplementary Figure 15. GCR1/2 are required for miR156-induced glandular trichome formation.

a Transgenic plants with *miR156B* overexpression. Bar: 10 cm. **b** SEM images show that *miR156B* overexpression induces DGT formation. Bar: 100 μm. **c and d** Expression of *miR156B* driven by *MTR1* promoter induces DGT formation. Relative expression level of *miR156B* in *pMTR1:miR156B* transgenic lines **(c)**. Data are shown as mean ± SD (n=3 biological replicates). *p*-values obtained by unpaired two-sided *t*-test. SEM images show DGT in *pMTR1:miR156B* transgenic line #3 **(d)**. Bar: 100 μm. **e** Quantification of trichomes in *35S:miR156B* and *pMTR1: miR156B*. The Y-axis represents the proportion of three categories of trichomes in the total number of trichomes. n represents the number of SEM images used for the quantification. At

least 15 different SEM images from three to four individual plants are used for trichome quantification. Data are shown as mean ± SD. *p*-values were calculated by unpaired two-sided *t*-test and presented in the Supplementary Table 10. **f** Relative expression level of *GCR1*, *GCR2* and *SlTOE1B* in *pMTR1: miR156B* lines. Data are shown as mean ± SD (n=3 biological replicates). *p*-values obtained by unpaired two-sided *t*-test.

a

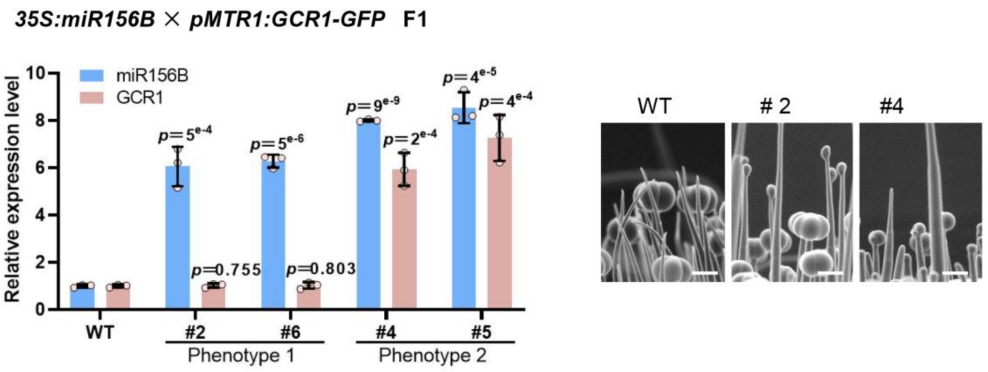

b

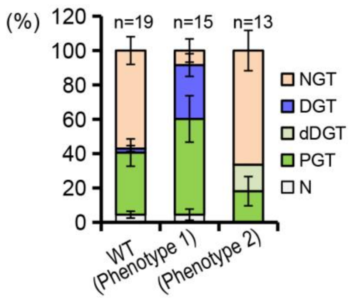

Supplementary Figure 16. GCR1 is epistatic to miR156B in the gland formation pathway.

a Gland development is inhibited in F1 plants of the cross between *pMTR1:GCR1-GFP* and *35S:miR156B*. Data are shown as mean ± SD (n=3 technical replicates). Exact *p*-values were calculated by unpaired two-sided *t*-test. Trichome phenotype of Line 2 (#2) and Line 6 (#6) is the same as *35S:miR156B* plants (Phenotype 1) and trichome phenotype of Line 4 (#4) and Line 5 (#5) is the same as *pMTR1: GCR1-GFP* plants (Phenotype 2). SEM images show trichomes of line 2 (#2) and line 4 (#4). Bar: 50 µm. **b** Quantification of trichomes on the adult stems of F1 plants shown in **(a).** n represents the number of SEM images used for the quantification. For each line, at least 13 different SEM images from three to five individual plants were used for trichome quantification. Data are shown as mean ± SD. *p*-values were calculated by unpaired two-sided *t*-test and presented in the Supplementary Table 11.

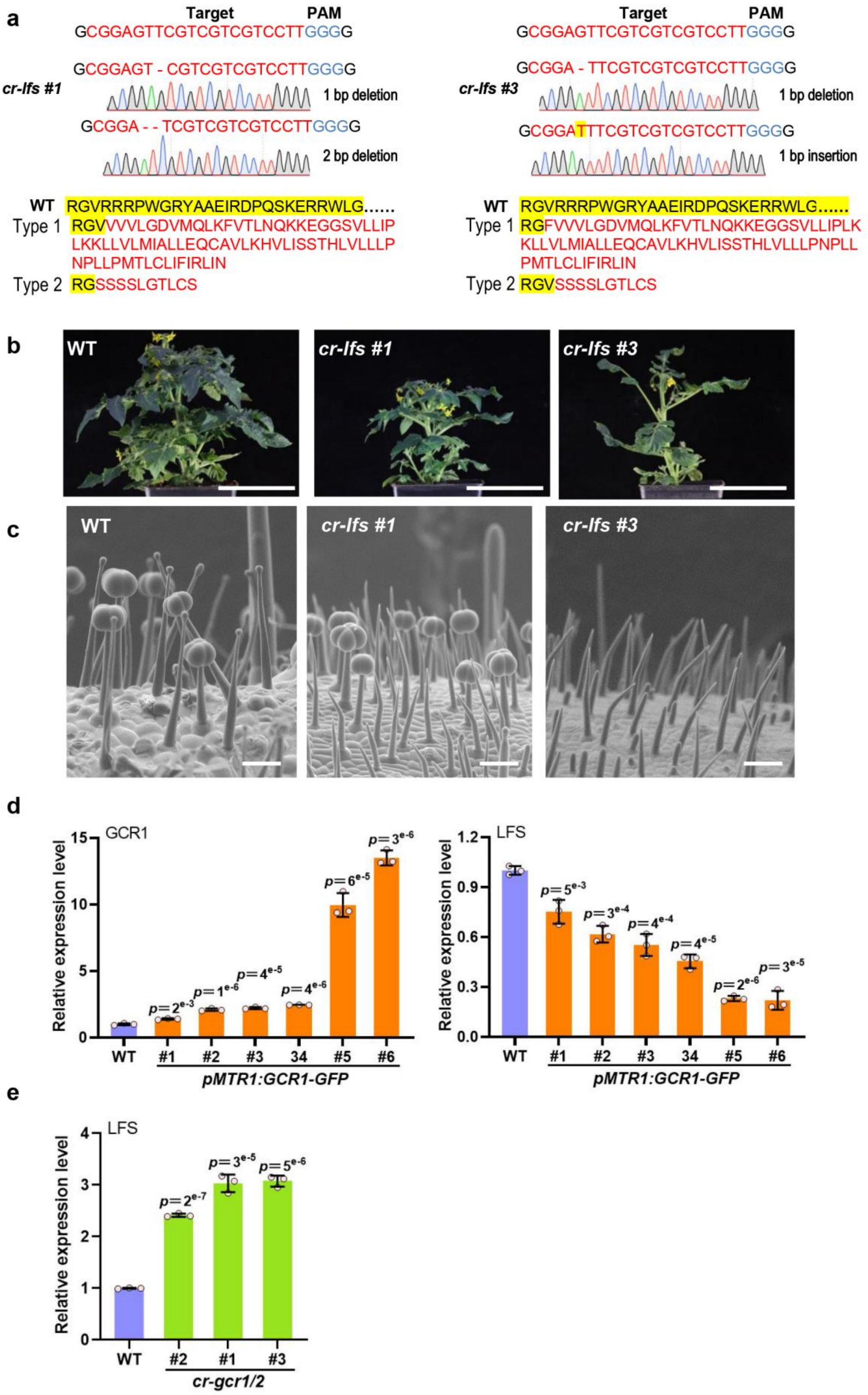

Supplementary Figure 17. Genotype and phenotype of *lfs* mutant.

a Knockout of *LFS* by CRISPR/Cas9 (*cr-lfs*). The red lines indicate the single guide

RNA target sequences. The sequences in blue indicate the protospacer-adjacent motif (PAM) sequences. Two knockout lines of LFS are shown: *cr-lfs* #1 and *cr-lfs* #3. *cr-lfs* #1 is a heterozygous mutant with a single DNA strand edited . Two types of mutations are generated in *cr-lfs* #1: type 1 with 1 base-pair deletion and type 2 with 2 base-pairs deletion, both of which result in the truncated proteins. All DNA strands of *cr-lfs* #3 are edited and two types of mutations are identified: type 1 with 1 base-pair deletion and type 2 with 1 base-pair deletion insertion, both of which result in the truncated proteins. **b** *lfs* mutant plants. Bar: 10 cm. **c** SEM images of juvenile stems show that knockout of *LFS* inhibits gland formation. Bar: 100 μm. **d** Relative expression level of *GCR1* and *LFS* in *pMTR1:GCR1-GFP* lines. Data are shown as mean ± SD (n=3 biological replicates). *p*-values obtained by unpaired two-sided *t*-test. **e** Relative expression level of *LFS* in *cr-gcr1/2* lines. Data are shown as mean ± SD (n=3 biological replicates). *p*-values obtained by unpaired two *t*-test.

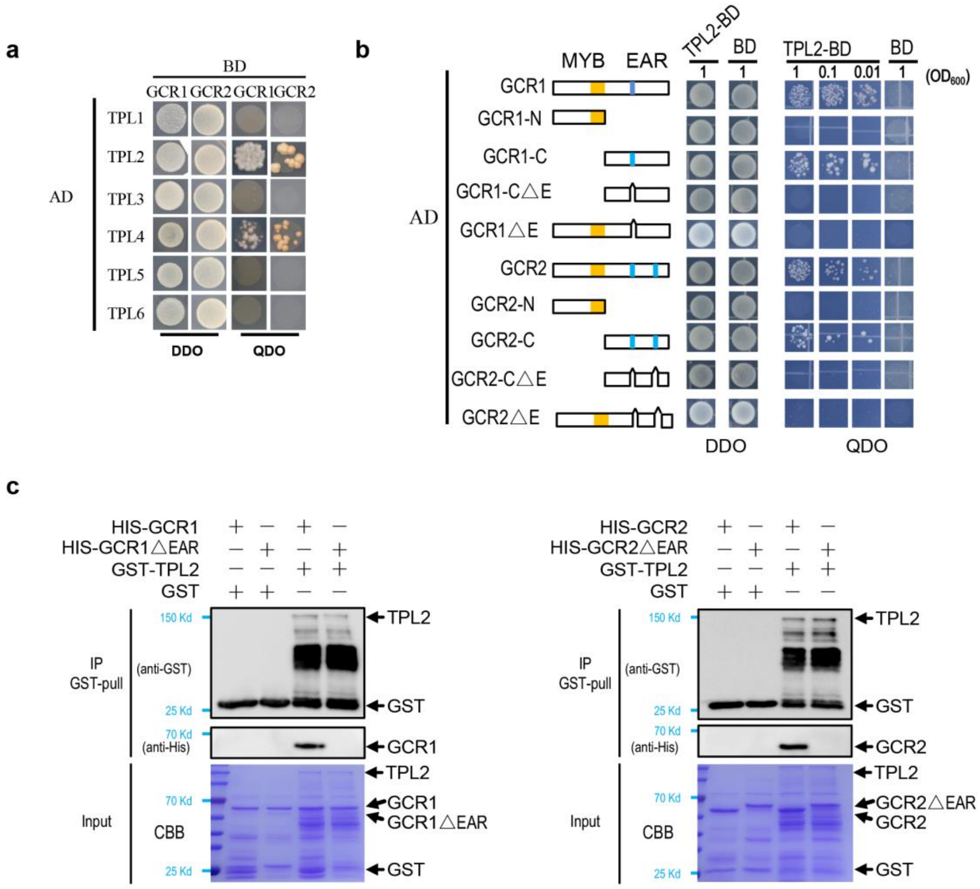

Supplementary Figure 18. GCR1/2 interact with TPL.

a Y2H assays show that GCR1/2 interact with TPL2 and TPL4. **b** Y2H assays show GCR1 and GCR2 interact with TPL2 by EAR motif. GCR1/2-N represents the N-terminal of GCR1/2; GCR1/2-C represents the C-terminal of GCR1/2; GCR1/2-C△E represents the C-terminal of GCR1/2 with EAR motif deletion. GCR1/2△E represents the full length of GCR1/2 with EAR motif deletion. DDO: SD/-Leu-Trp. QDO: SD/-Ade-His-Leu-Trp. Three dilutions for each interaction are presented. **c** GST-pull down assay. HIS-GCR1/2 with EAR motif deletion is marked as HIS-GCR1△EAR and HIS-GCR2△EAR. GFP (left two lanes; negative control) or GFP fused with the target protein (the other lanes) are used as bait to bind to the GFP beads. Immuno-precipitated proteins are detected by anti-HIS antibody. Data represent results of three independent experiments including Y2H assays and GST-pull down assays.

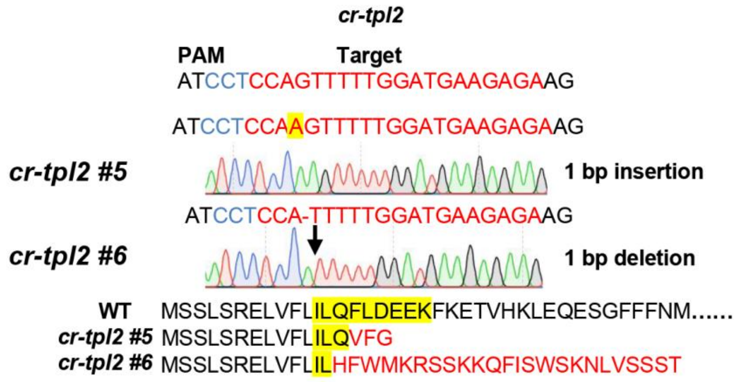

Supplementary Figure 19. Sequence analysis of *tpl2* mutant generated by CRISPR/Cas9.

The red lines indicate the single guide RNA target sequences. The sequences in blue indicate the protospacer-adjacent motif (PAM) sequences. Two knockout lines of TPL2 are shown: *cr-tpl2* #5 and #6. There are 1 base-pair insertion in *cr-tpl2* #5 and 1 base-pair deletion in *cr-tpl2* #6 which result in the truncated proteins.

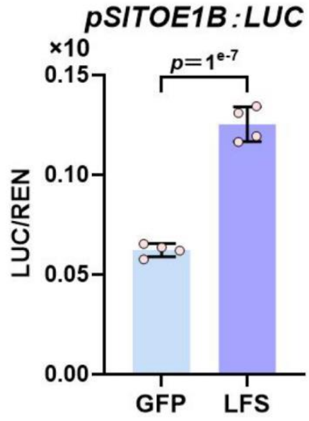

Supplementary Figure 20. LUC assay shows LFS activates the transcriptional activity of *SlTOE1B* promoters in tobacco protoplasts.

n = 4 biological replicates. *p*-values were calculated by unpaired two-sided *t*-test.

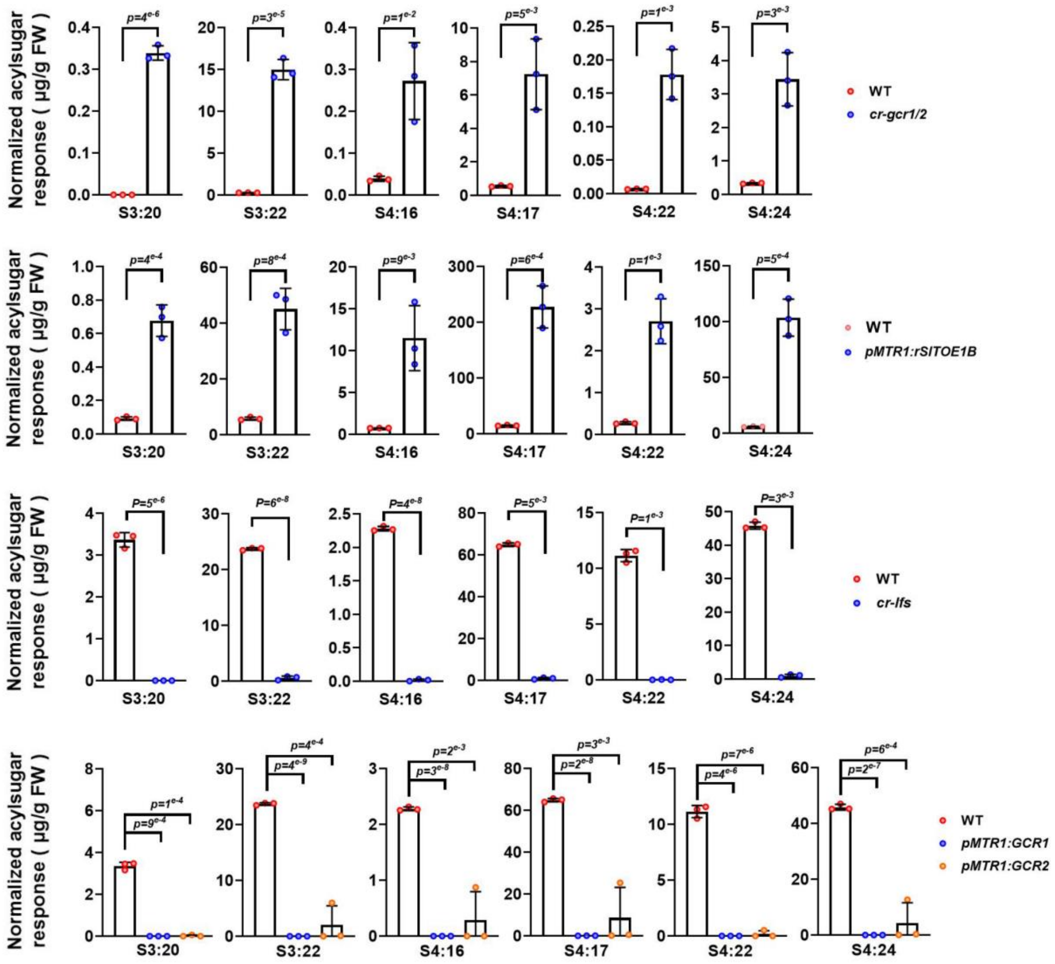

Supplementary Figure 21. Measurement of acylsugars by LC-MS according to the previously published method.

Data are shown as mean ± SD (n=3 biological replicates). *p*-values obtained by unpaired two-sided *t*-test.

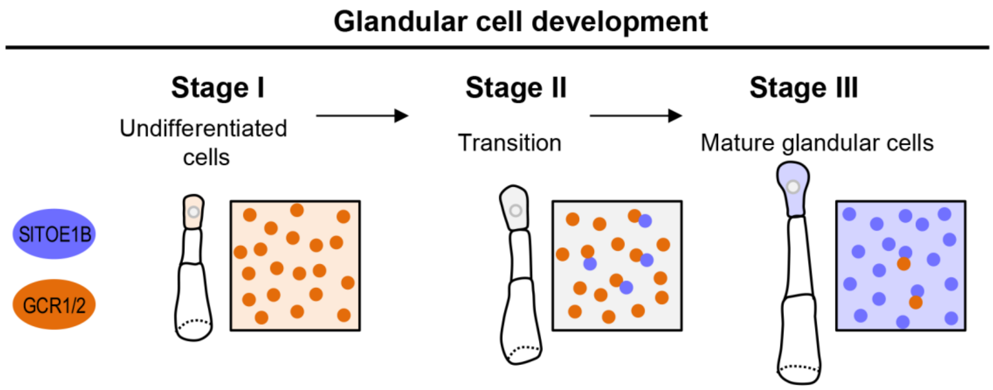

Supplementary Figure 22. A putative model depicting the coordination of GCR1/2 and SlTOE1B during glandular cell formation.

Supplementary Table 1. Annotation of positive clones in Y2H screening using GCR1 as a bait.

**Gene ID**	**Number of positive clone**	**Percentage of total positive clones**	**Protein name**	**Homolog of Arabidopsis**	**Annotation**
Solyc04g049800	42	32.81%	TOE1	AT2G28550	AP2 family transcription factor
Solyc03g110860	3	2.34%	NF-YC1	AT3G48590	Nuclear transcription factor Y subunit C-1
Solyc06g072040	2	1.56%	*CIP2a*	AT3G48590	Nuclear transcription factor Y subunit C-1
Solyc04g077490	1	0.78%	ANT	AT4G37750	A putative transcriptional regulator similar to AP2
Solyc10g079070	1	0.78%	*SlbHLH065*	AT3G57800	Regulate hypocotyl elongation
Solyc01g008230	1	0.78%	*SlTCP15*	AT5G23280	Involved in leaf development and axillary shoots formation.
Solyc02g085950	18	14.06%	RBCS3B	AT5G38410	Rubisco small subunit (RBCS) multigene family
Solyc03g034220	10	7.81%	Slss5	AT5G38410	Rubisco small subunit (RBCS) multigene family
Solyc12g005630	8	6.25%	PETC, PGR1	AT4G03280	Cytochrome b6-f complex iron-sulfur subunit
Solyc10g086580	5	3.91%		AT2G39730	Ribulose-1 5-bisphosphate carboxylase/oxygenase activase 1
Solyc01g105050	4	3.13%		AT1G15820	Chlorophyll a-b binding protein
Solyc01g096150	2	1.56%		AT1G01225	NC domain-containing protein
Solyc01g097010	2	1.56%		AT3G04610	Nucleic acid binding protein
Solyc04g015020	2	1.56%	ATHMP35	AT4G16380	Proline-rich protein
Solyc06g083620	2	1.56%		AT4G29040	26S protease regulatory subunit 4
Solyc08g008470	2	1.56%			
Other clones	23	17.97%			

Supplementary Table 2. P-value of quantification of trichomes in figure 1e.

	NGT		
	Average	SD	P-Value
WT	76.74854	3.20844	
cr-gcr1	56.08000	13.51929	0.00144, ** (*cr-gcr1* vs WT)
cr-gcr2	75.19709	3.39759	0.1939, (*cr-gcr2* vs WT)
cr-gcr1/2	4.27852	1.65451	2.73262^{e-32}, *** (*cr-gcr1/2* vs WT)<br>1.70071^{e-13}, *** (*cr-gcr1/2* vs *cr-gcr1*)<br>4.27538^{e-31}, *** (*cr-gcr1/2* vs *cr-gcr2*)
	DGT		
	Average	SD	P-Value
WT	0.00000	0.00000	
cr-gcr1	26.63346	9.62693	4.29132^{e-12}, *** (*cr-gcr1* vs WT)
cr-gcr2	0.00000	0.00000	
cr-gcr1/2	79.80067	2.36857	4.38918^{e-42}, *** (*cr-gcr1/2* vs WT)<br>6.74871^{e-17}, *** (*cr-gcr1/2* vs *cr-gcr1*)<br>2.39097^{e-37}, *** (*cr-gcr1/2* vs *cr-gcr2*)
	PGT		
	Average	SD	P-Value
WT	23.25146	3.20844	
cr-gcr1	17.28653	5.59106	0.00092, *** (*cr-gcr1* vs WT)
cr-gcr2	24.80291	3.39759	0.22999, (*cr-gcr2* vs WT)
cr-gcr1/2	15.92081	2.14219	4.12274^{e-08}, *** (*cr-gcr1/2* vs WT)<br>0.40568, (*cr-gcr1/2* vs *cr-gcr1*)<br>1.14870^{e-08}, *** (*cr-gcr1/2* vs *cr-gcr2*)

p-values were obtained by unpaired two-sided *t*-test.

Supplementary Table 3. P-value of quantification of trichomes in figure 2d.

	NGT		
	Average	SD	P-Value
WT	1.93699	2.66827	
pMTR1:GCR1	51.09046	9.08866	7.04127^{e-23}, *** (*pMTR1:GCR1* vs WT)
pMTR1:GCR2	52.54066	8.02333	2.92651^{e-24}, *** (*pMTR1:GCR2* vs WT)
pMTR1:NbGCR	45.91640	5.43791	2.45399^{e-24}, *** (*pMTR1:NbGCR1* vs WT)
pMTR1:PeGCR	46.47423	5.79386	4.09252^{e-17}, *** (*pMTR1:PeGCR1* vs WT)
	DGT		
	Average	SD	P-Value
WT	45.56827	11.16015	
pMTR1:GCR1	0.00000	0.00000	1.00647^{e-16}, *** (*pMTR1:GCR1* vs WT)
pMTR1:GCR2	0.00000	0.00000	5.52419^{e-16}, *** (*pMTR1:GCR2* vs WT)
pMTR1:NbGCR	0.00000	0.00000	1.10232^{e-13}, *** (*pMTR1:NbGCR1* vs WT)
pMTR1:PeGCR	0.00000	0.00000	1.00647^{e-16}, *** (*pMTR1:PeGCR1* vs WT)
	dPGT		
	Average	SD	P-Value
WT	0.00000	0.00000	
pMTR1:GCR1	25.67144	4.62728	3.76427^{e-24}, *** (*pMTR1:GCR1* vs WT)
pMTR1:GCR2	25.77243	5.19004	3.33244^{e-22}, *** (*pMTR1:GCR2* vs WT)
pMTR1:NbGCR	33.98648	5.90426	1.99334^{e-16}, *** (*pMTR1:NbGCR1* vs WT)
pMTR1:PeGCR	32.78665	4.67172	1.97684^{e-26}, *** (*pMTR1:PeGCR1* vs WT)
	PGT		
	Average	SD	P-Value
WT	49.54519	8.96585	
pMTR1:GCR1	21.14755	5.48796	2.44081^{e-12}, *** (*pMTR1:GCR1* vs WT)
pMTR1:GCR2	19.07359	5.53306	1.29389^{e-12}, *** (*pMTR1:GCR2* vs WT)
pMTR1:NbGCR	17.74634	3.47535	4.49866^{e-13}, *** (*pMTR1:NbGCR1* vs WT)
pMTR1:PeGCR	18.94579	6.08689	6.21863^{e-12}, *** (*pMTR1:PeGCR1* vs WT)
	N		
	Average	SD	P-Value
WT	2.91798	2.69085	
pMTR1:GCR1	2.09055	2.25785	0.34628, (*pMTR1:GCR1* vs WT)
pMTR1:GCR2	2.61331	2.05595	0.72757, (*pMTR1:GCR2* vs WT)
pMTR1:NbGCR	2.35078	1.52178	0.45200, (*pMTR1:NbGCR1* vs WT)
pMTR1:PeGCR	0.01793	0.02184	0.00079, *** (*pMTR1:PeGCR1* vs WT)

p-values were obtained by unpaired two-sided *t*-test.

Supplementary Table 4. P-value of quantification of trichomes in figure 4e.

	NGT		
	Average	SD	P-Value
WT	64.80523	5.55099	
pMTR1:rSlTOE1B	0.00000	0.00000	1.14848^{e-52}, *** (*pMTR1:rSlTOE1B* vs WT)
	DGT		
	Average	SD	P-Value
WT	1.57385	2.50852	
pMTR1:rSlTOE1B	58.13140	6.51422	7.04748^{e-41}, *** (*pMTR1:rSlTOE1B* vs WT)
	PGT		
	Average	SD	P-Value
WT	25.68163	2.82098	
pMTR1:rSlTOE1B	35.45805	7.44657	1.93096^{e-07}, *** (*pMTR1:rSlTOE1B* vs WT)
	N		
	Average	SD	P-Value
WT	7.93928	4.91125	
pMTR1:rSlTOE1B	7.26955	4.17810	0.31868, (*pMTR1:rSlTOE1B* vs WT)

p-values were obtained by unpaired two-sided *t*-test.

Supplementary Table 5. P-value of quantification of trichomes in figure 4g.

	NGT		
	Average	SD	P-Value
WT	8.02515	7.74626	
cr-sltoe1b	18.25261	10.68665	0.00170, *** (*cr-sltoe1b vs*WT)
	DGT		
	Average	SD	P-Value
WT	19.58280	8.22352	
cr-sltoe1b	8.22430	3.08835	0.00021, *** (*cr-sltoe1b* vs WT)
	PGT		
	Average	SD	P-Value
WT	69.73267	7.84332	
cr-sltoe1b	72.24430	13.55013	0.38353, (*cr-sltoe1b* vs WT)
	N		
	Average	SD	P-Value
WT	2.65939	3.48240	
cr-sltoe1b	1.27878	1.46680	0.22268, (*cr-sltoe1b* vs WT)

p-values were obtained by unpaired two-sided *t*-test.

Supplementary Table 6. P-value of quantification of trichomes in figure 5b.

	NGT		
	Average	SD	P-Value
WT	53.50468	10.06659	
pMTR1:rSlTOE1B	0.00000	0.00000	1.30491^{e-32}, *** (*pMTR1:rSlTOE1B* vs WT)
pMTR1:GCR1	74.99150	9.65256	1.18776^{e-07}, *** (*pMTR1:GCR1* vs WT)<br>1.15134^{e-39}, *** (*pMTR1:GCR1* vs *pMTR1:rSlTOE1B*)
	DGT		
	Average	SD	P-Value
WT	0.63989	1.13032	
pMTR1:rSlTOE1B	57.35540	7.23384	4.03145^{e-35},*** (*pMTR1:rSlTOE1B* vs WT)
pMTR1:GCR1	0.00000	0.00000	0.02185, (*pMTR1:GCR1* vs WT)<br>1.89038^{e-34}, *** (*pMTR1:GCR1* vs *pMTR1:rSlTOE1B*)
	dPGT		
	Average	SD	P-Value
WT	0.00000	0.00000	
pMTR1:rSlTOE1B	0.00000	0.00000	
pMTR1:GCR1	14.53673	4.53742	6.92987^{e-16}, *** (*pMTR1:GCR1* vs WT)<br>1.06967^{e-22}, *** (*pMTR1:GCR1* vs *pMTR1:rSlTOE1B*)
	PGT		
	Average	SD	P-Value
WT	35.61300	6.32415	
pMTR1:rSlTOE1B	33.39627	5.50705	0.19788, (*pMTR1:rSlTOE1* vs WT)
pMTR1:GCR1	10.47177	7.48332	5.70469^{e-13}, *** (pMTR1:GCR1 vs WT)<br>2.74430^{e-16}, *** (*pMTR1:GCR1* vs *pMTR1:rSlTOE1B*)
	N		
	Average	SD	P-Value
WT	10.15373	6.86552	
pMTR1:rSlTOE1B	9.24833	4.93418	0.64042, (*pMTR1:rSlTOE1B* vs WT)
pMTR1:GCR1	0.00000	0.00000	3.41059^{e-07}, *** (*pMTR1:GCR1* vs WT)<br>3.39005^{e-10}, *** (*pMTR1:GCR1* vs *pMTR1:rSlTOE1B*)

p-values were obtained by unpaired two-sided *t*-test.

Supplementary Table 7. P-value of quantification of trichomes in figure 8b.

	NGT		
	Average	SD	P-Value
WT	87.47876	3.65358	
cr-lfs	100.00000	0.00000	1.91693^{e-13}, *** (*cr-lfs* vs WT)
cr-gcr1/2	0.00000	0.00000	6.93438^{e-40}, *** (*cr-gcr1/2* vs WT)
cr-gcr1/2×cr-lfs	100.00000	0.00000	1.59019^{e-16}, ***(*cr-gcr1/2×cr-lfs* vs WT)
	DGT		
	Average	SD	P-Value
WT	0.00000	0.00000	
cr-lfs	0.00000	0.00000	
cr-gcr1/2	92.84777	5.43531	2.18511^{e-38}, *** (*cr-gcr1/2* vs WT)
cr-gcr1/2×cr-lfs	0.00000	0.00000	4.38918^{e-42}, ***(*cr-gcr1/2×cr-lfs* vs WT)<br>2.18512^{e-38}, *** (*cr-gcr1/2×cr-lfs* vs *cr-gcr1/2*)
	PGT		
	Average	SD	P-Value
WT	12.52124	3.65358	
cr-lfs	0.00000	0.00000	1.91693^{e-13}, *** (*cr-lfs* vs WT)
cr-gcr1/2	7.15223	5.43531	0.00214, ** (*cr-gcr1/2* vs WT)
cr-gcr1/2×cr-lfs	0.00000	0.00000	1.59019^{e-16}, ***(*cr-gcr1/2×cr-lfs* vs WT)<br>2.00219^{e-06}, *** (*cr-gcr1/2×cr-lfs* vs *cr-gcr1/2*)

p-values were obtained by unpaired two-sided *t*-test.

Supplementary Table 8. P-value of quantification of trichomes in figure S1b.

	NGT		
	Average	SD	P-Value
AC	15.41000	2.98915	
S. Pennellii	0.00000	0.00000	6.11548^{e-28} *** (*S. Pennellii* vs AC)
	DGT		
	Average	SD	P-Value
AC	27.61137	6.98275	
S. Pennellii	98.40385	1.90934	1.78130^{e-37}, *** (S. Pennellii vs AC)
	PGT		
	Average	SD	P-Value
AC	56.97863	8.06392	
S. Pennellii	1.59615	1.90934	2.14884^{e-31}, *** (S. Pennellii vs AC)

p-values were obtained by unpaired two-sided *t*-test.

Supplementary Table 9. P-value of quantification of trichomes in figure S11c.

	NGT		
	Average	SD	P-Value
WT	3.69006	2.54926	
cr-sltoe1b	28.74175	10.42325	1.46897^{e-06}, *** (*cr-sltoe1b* vs WT)
cr-sltoe1b/cr-gcr1	0.00000	0.00000	0.00029, *** (*cr-sltoe1b/cr-gcr1* vs WT)<br>1.07797^{e-08}, *** (*cr-sltoe1b/cr-gcr1* vs *cr-sltoe1b*)
	DGT		
	Average	SD	P-Value
WT	26.69287	3.67950	
cr-sltoe1b	4.23503	4.04797	1.51363^{e-11}, *** (*cr-sltoe1b* vs WT)
cr-sltoe1b/cr-gcr1	33.40297	5.24900	0.00754, ** (*cr-sltoe1b/cr-gcr1* vs WT)<br>$8.97323^{e-14,}$*** (*cr-sltoe1b/cr-gcr1* vs *cr-sltoe1b*)
	PGT		
	Average	SD	P-Value
WT	65.22112	4.47421	
cr-sltoe1b	61.52907	6.47896	0.24348, (*cr-sltoe1b* vs WT)
cr-sltoe1b/cr-gcr1	63.63138	5.98381	0.54178, (*cr-sltoe1b/cr-gcr1* vs WT)<br>0.59267, (*cr-sltoe1b/cr-gcr1* vs *cr-sltoe1b*)
	N		
	Average	SD	P-Value
WT	4.39595	2.17300	
cr-sltoe1b	5.49416	4.40600	0.51744, (*cr-sltoe1b* vs WT)
cr-sltoe1b/cr-gcr1	2.96565	1.78862	0.14464, (*cr-sltoe1b/cr-gcr1* vs WT)<br>0.10011, (*cr-sltoe1b/cr-gcr1* vs *cr-sltoe1b*)

p-values were obtained by unpaired two-sided *t*-test.

Supplementary Table 10. P-value of quantification of trichomes in figure S15e.

	NGT		
	Average	SD	P-Value
WT	69.20174	6.99667	
35S:miR156B	0	0	3.36490^{e-36}, *** (*35S:miR156B* vs WT)
pMTR1:miR156B	3.75435	3.52980	1.03779^{e-28}, *** (*pMTR1:miR156B* vs WT)
	DGT		
	Average	SD	P-Value
WT	0.50627	0.84314	
35S:miR156B	48.24196	7.58475	1.55470^{e-29}, *** (*35S:miR156B* vs WT)
pMTR1:miR156B	31.14024	5.85880	2.90898^{e-24}, *** (*pMTR1:miR156B* vs WT)
	PGT		
	Average	SD	P-Value
WT	27.96025	5.34091	
35S:miR156B	51.71338	7.59273	5.58875^{e-15}, *** (*35S:miR156B* vs WT)
pMTR1:miR156B	62.90222	5.94822	3.07086^{e-20}, *** (*pMTR1:miR156B* vs WT)
	N		
	Average	SD	P-Value
WT	2.33172	2.80952	
35S:miR156B	0.04464	0.19964	0.00079, ** (****S:miR156B* vs WT)
pMTR1:miR156B	2.20316	2.47396	0.88605, (*pMTR1:miR156B* vs WT)

p-values were obtained by unpaired two-sided *t*-test.

Supplementary Table 11. P-value of quantification of trichomes in figure S16b.

	NGT		
	Average	SD	P-Value
WT	57.05781	8.06261	
35S:miR156B	8.43720	6.78074	8.72100^{e-19}, *** (*35S:miR156B* vs WT)
pMTR1:GCR1	66.43301	11.73391	0.01359, * (*pMTR1:GCR1* vs WT)<br>7.37283^{e-15}, *** (*pMTR1:GCR1* vs *35S:miR156B*)
	DGT		
	Average	SD	P-Value
WT	2.32611	1.74707	
35S:miR156B	31.35628	6.60291	1.32535^{e-18}, *** (*35S:miR156B* vs WT)
pMTR1:GCR1	0	0	0.00004, *** (*pMTR1:GCR1* vs WT)<br>1.19211^{e-15}, *** (*pMTR1:GCR1* vs *35S:miR156B*)
	dPGT		
	Average	SD	P-Value
WT	0	0	
35S:miR156B	0	0	(*35S:miR156B* vs WT)
pMTR1:GCR1	15.43239	5.51496	8.30439^{E-13}, *** (*pMTR1:GCR1* vs WT)<br>8.52421^{E-11}, *** (*pMTR1:GCR1* vs *35S:miR156B*)
	PGT		
	Average	SD	P-Value
WT	36.15156	7.95586	
35S:miR156B	55.73454	13.53319	9.04981^{e-06}, *** (*35S:miR156B* vs WT)
pMTR1:GCR1	18.13459	8.50002	1.34237^{e-06}, *** (*pMTR1:GCR1* vs WT)<br>4.99305^{e-09}, *** (*pMTR1:GCR1* vs *35S:miR156B*)
	N		
	Average	SD	P-Value
WT	4.46450	1.98304	
35S:miR156B	4.47196	3.24916	0.99346, (*35S:miR156B* vs WT)
pMTR1:GCR1	0	0	5.16256^{e-09}, *** (*pMTR1:GCR1* vs WT)<br>0.00004, *** (*pMTR1:GCR1* vs *35S:miR156B*)

p-values were obtained by unpaired two-sided *t*-test.

Supplementary Table 12. Primers used in this study.

Analysis	Primer name	Sequence(5' to 3')	Usage
**Vector Construction**	CR-GCR1-F	GAATCTAACAGTGTAGTTTGCAACAAAGGGAAACTCGAAGGTTTTAGAGCTAGAAATAG	*gcr1* mutant
	CR-GCR1-R	GCTATTTCTAGCTCTAAAACACGTAGCCTTGGCAATTTTGCAAACTACACTGTTAGATT	
	CR-GCR2-F	GAATCTAACAGTGTAGTTTGTCAGGAGTCCATCTTAGCCTGTTTTAGAGCTAGAAATAG	*gcr2* mutant
	CR-GCR2-R	GCTATTTCTAGCTCTAAAACAGGCTAAGATGGACTCCTGACAAACTACACTGTTAGATT	
	CR-SlTPL2-F	GAATCTAACAGTGTAGTTTGTCTCTTCATCCAAAAACTGGGTTTTAGAGCTAGAAATAG	*sltpl2* mutant
	CR-SlTPL2-R	GCTATTTCTAGCTCTAAAACCCAGTTTTTGGATGAAGAGACAAACTACACTGTTAGATT	
	CR-SlTOE1B-F	GAATCTAACAGTGTAGTTTGTGTCTGTGTCGGAATCCGCTGTTTTAGAGCTAGAAATAG	*Sltoe1b* mutant
	CR-SlTOE1B-R	GCTATTTCTAGCTCTAAAACGAGGTTTGTAAACAGGCACTCAAACTACACTGTTAGATT	
	35S-miR156b-F	CATTTGGAGAGGACACGCTCGAGGACAAAACATCATGTCTCTCCATT	*35S:miR156B*
	35S-miR156b-R	TCTCATTAAAGCAGGACTCTAGACAAACTCAAACGCAAAGACTTTTC	
	35S-GCR1-F	CATTTGGAGAGGACACGCTCGAGATGGAAAATGCATCTTCTTCATCCA	*35S:GCR1*
	35S-GCR1-R	TCGCCCTTGCTCACCATGAATTCAAGAGAAAGCATTGTGTTTATATCT	
	pMTR1-F	TGCATCCAACGCGTTGGGAGCTCCAGGTCGTCGCAAGGTTC	*pMTR1:GCR1*
	pMTR1-GCR1-R	GAAGATGCATTTTCCATCTCGAGCTAAAATCAAACAAAATAAAAAATA	
	pMTR1-GCR2-F	TTTGATTTTAGGAGCTCCTCGAGATGACAAGTGATTCCATTGATCTGA	*pMTR1:GCR2*
	pMTR1-GCR2-R	AGCTTGGTACCGGTACCCTCGAGGGTTGCACTTGATGAATAAGAAGGC	
	pMTR1-miR156b-F	TTTGATTTTAGGAGCTCCTCGAGGACAAAACATCATGTCTCTCCATT	*pMTR1:miR156B*
	pMTR1-miR156b-R	AGCTTGGTACCGGTACCCTCGAGCAAACTCAAACGCAAAGACTTTTC	
	pMTR1- NbGCR-F	TTTGATTTTAGGAGCTCCTCGAGATGATTCATATAGGCTTGTTTCT	*pMTR1:NbGCR*
	pMTR1- NbGCR-R	AGCTTGGTACCGGTACCCTCGAGGGTTGCACTTGATGAATAATTTGGC	
	pMTR1- PeGCR-F	TTTGATTTTAGGAGCTCCTCGAGATGGAATACATTACGGGAAAAGCCT	*pMTR1:PeGCR*
	pMTR1- PeGCR-R	AGCTTGGTACCGGTACCCTCGAGCAAGTACTCAGCCTCCTCTCTAGGC	
	pMTR1- rSlTOE1B-F1	TTTGATTTTAGGAGCTCCTCGAGATGTTGGATCTGAATAATGATAG	*pMTR1:* rSlTOE1B
	pMTR1- rSlTOE1B-R1	AAAAGCCGGACGAGGCCGCGGAAGAAGAAAATACTGGCACTGGA	
	pMTR1- rSlTOE1B-F2	TCCGCGGCCTCGTCCGGCTTTTCTACTGCTACCACTCTGCATAC	
	pMTR1- rSlTOE1B-R2	AGCTTGGTACCGGTACCCTCGAGGTTGGTGGGGAGACGTAGTTG	
	pGCR1-nStayGold-F	TGCATCCAACGCGTTGGGAGCTCGGATTATTTAGTGGCAAGTTTG	*pGCR1:NLS-Staygold*
	pGCR1-nStayGold-R	AGCTTGGTACCGGTACCCTCGAGGACTAATTATACGTCTTGTC	
	pGCR2-nStaygold-F	TGCATCCAACGCGTTGGGAGCTCTCCGAAAAAGAAAACTCCAAAAG	*pGCR2:NLS-Staygold*
	pGCR2-nStaygold-R	AGCTTGGTACCGGTACCCTCGAGGATTATTATTGATGCAAGAACAAATCCTG	
	pSlTOE1B-nVenus-F	TGCATCCAACGCGTTGGGAGCTCTTTTTCTAGTATTGGTTGGCAGTGG	*pSlTOE1B:NLS-Vneus*
	pSlTOE1B-nVenus-R	AGCTTGGTACCGGTACCCTCGAGGCTCCGTTTTTTGCAAATTATTAC	
**Y2H**	GCR1-AD-F	GTACCAGATTACGCTCATATGATGGAAAATGCATCTTCTTCATCCA	GCR1-AD and GCR1ΔE-AD
	GCR1-AD-R	CAGCTCGAGCTCGATGGATCCAAGAGAAAGCATTGTGTTTATATCT	
	GCR2-AD-F	GTACCAGATTACGCTCATATGATGACAAGTGATTCCATTGATCTGA	GCR2-AD and GCR2ΔE-AD
	GCR2-AD-R	CAGCTCGAGCTCGATGGATCCGGTTGCACTTGATGAATAAGAAGGC	
	SlTOE1B-AD-F	GTACCAGATTACGCTCATATGATGTTGGATCTGAATAATGATAGTG	SlTOE1B-AD
	SlTOE1B-AD-R	CAGCTCGAGCTCGATGGATCCTTAGTTGGTGGGGAGACGTAGTTGGTTG	
	SlTPL1-AD-F	GTACCAGATTACGCTCATATGATGTCATCTCTCAGTAGAGA	SlTPL1-AD
	SlTPL1-AD-R	CAGCTCGAGCTCGATGGATCCTCATCTTGGTGCTTGATCGG	
	SlTPL2-AD-F	GTACCAGATTACGCTCATATGATGTCTTCCTTGAGTAGGGA	SlTPL2-AD
	SlTPL2-AD-R	CAGCTCGAGCTCGATGGATCCTCACCTTGAAGGTGTTTCTG	
	SlTPL3-AD-F	GTACCAGATTACGCTCATATGATGTCTTCTCTTAGCAGAGA	SlTPL3-AD
	SlTPL3-AD-R	CAGCTCGAGCTCGATGGATCCTCATCTTTGAACTTGGTCAG	
	SlTPL4-AD-F	GTACCAGATTACGCTCATATGATGACTTCTTTAAGCAGAGA	SlTPL4-AD
	SlTPL4-AD-R	CAGCTCGAGCTCGATGGATCCCTACCTTGATGCTTGATCAA	

	SlTPL5-AD-F	GTACCAGATTACGCTCATATGATGAGGCATTTTGATGAAAT	SlTPL5-AD
	SlTPL5-AD-R	CAGCTCGAGCTCGATGGATCCCTACCTTTGAGGTTGATCTG	
	SlTPL6-AD-F	GTACCAGATTACGCTCATATGATGTCTCTTAGTAAGGACCT	SlTPL6-AD
	SlTPL6-AD-R	CAGCTCGAGCTCGATGGATCCCTATATTGGTTGCTCATTGG	
	GCR1-N-AD-F	GTACCAGATTACGCTCATATGATGGAAAATGCATCTTCTTC	GCR1-N-AD
	GCR1-N-AD-R	CAGCTCGAGCTCGATGGATCCTCATTGTCCAGATTCATCAA	
	GCR1-C-AD-F	GTACCAGATTACGCTCATATGATGGTGTTAGGGCATGGTAAT	GCR1-C-AD
	GCR1-C-AD-R	CAGCTCGAGCTCGATGGATCCCTAAAGAGAAAGCATTG	
	GCR1-C△E-AD-R	GAAAAAGAATGGTTACCTGATAGCAGAAGCTCAGATGACAAG	GCR1-C△E-AD
	GCR1-C△E-AD-F	CTTGTCATCTGAGCTTCTGCTATCAGGTAACCATTCTTTTTC	
	GCR2-N-AD-F	GTACCAGATTACGCTCATATGATGACAAGTGATTCCATTG	GCR2-N-AD
	GCR2-N-AD-R	CAGCTCGAGCTCGATGGATCCTTATACTTGTCCAAATTCTC	
	GCR2-C-AD-F	GTACCAGATTACGCTCATATGATGCTAGGCCATGCTTATAGAAC	GCR2-C-AD
	GCR2-C-AD-R	CAGCTCGAGCTCGATGGATCCTTAGGTTGCACTTGATGAATAAG	
	GCR2-C△E-AD-R1	CTATTATTATTGTCTTGATTAGTTGTAGGCAGCAAATAATCTTTTTC	GCR2-C△E-AD
	GCR2-C△E-AD-F2	GAAAAAGATTATTTGCTGCCTACAACTAATCAAGACAATAATAATAG	
	GCR2-C△E-AD-R2	CAGCTCGAGCTCGATGGATCCTTAGGTTGCACTTGATGAATAAGAAGTGTTTATATCTG	
	GCR1-BD-F	TCAGAGGAGGACCTGCATATGATGGAAAATGCATCTTCTTCATCCA	GCR1-BD
	GCR1-BD-R	CTAGTTATGCGGCCGCTGCAGAAGAGAAAGCATTGTGTTTATATCT	
	GCR2-BD-F	TCAGAGGAGGACCTGCATATGATGACAAGTGATTCCATTGATCTGA	GCR2-BD
	GCR2-BD-R	CTAGTTATGCGGCCGCTGCAGGGTTGCACTTGATGAATAAGAAGGC	
	SlTOE1B-BD-F	TCAGAGGAGGACCTGCATATGATGTTGGATCTGAATAATGATAGTG	SlTOE1B-BD
	SlTOE1B-BD-R	CTAGTTATGCGGCCGCTGCAGCTAGTTGGTGGGGAGACGTA	
	SlTOE1B-C-DE-BD-F	TCAGAGGAGGACCTGCATATGATGGAGAATTGGATTTCTCTGCC	SlTOE1B-C-△EAR -BD
	SlTPL2-BD-F	TCAGAGGAGGACCTGCATATGATGTCTTCCTTGAGTAGGG	SlTPL2-BD
	SlTPL2-BD-R	CTAGTTATGCGGCCGCTGCAGTCACCTTGAAGGTGTTTCTG	
	SlTOE1B-DE-BD-F	TCAGAGGAGGACCTGCATATGATGGATAGTGTTTTTTTCAAGG	SlTOE1B-△EAR-BD
	SlTOE1B-DE-BD-R	CTAGTTATGCGGCCGCTGCAGGTTGGTGGGGAGACGTAGTTGGTT	
**BIFC**	GCR1-2YN-F	CATTTACGAACGATAGTTAATTAACATGGAAAATGCATCTTCTTC	GCR1-nYFP and GCR1△EAR-nYFP
	GCR1-2YN-R	CCACCTCCTCCACTAGTGGCGCGCCCAAGAGAAAGCATTGTG	
	GCR1-N-2YN-F	CATTTACGAACGATAGTTAATTAACATGGAAAATGCATCTTCTTC	GCR1-N-nYFP
	GCR1-N-2YN-R	CCACCTCCTCCACTAGTGGCGCGCCCTTGTCCAGATTCATCAA	
	GCR1-C-2YN-F	CATTTACGAACGATAGTTAATTAACATGGTGTTAGGGCATGGTAAT	GCR1-C-nYFP
	GCR1-C-2YN-R	CCACCTCCTCCACTAGTGGCGCGCCCAAGAGAAAGCATTGTG	
	GCR2-2YN-F	CATTTACGAACGATAGTTAATTAACATGACAAGTGATTCCATTG	GCR2-nYFP and GCR2△EAR-nYFP
	GCR2-2YN-R	CCACCTCCTCCACTAGTGGCGCGCCCGGTTGCACTTGATGAATAAG	
	GCR2-N-2YN-F	CATTTACGAACGATAGTTAATTAACATGACAAGTGATTCCATTG	GCR2-N-nYFP
	GCR2-N-2YN-R	CCACCTCCTCCACTAGTGGCGCGCCCTACTTGTCCAAATTCTC	
	GCR2-C-2YN-F	CATTTACGAACGATAGTTAATTAACATGCTAGGCCATGCTTATAGAAC	GCR2-C-nYFP
	GCR2-C-2YN-R	CCACCTCCTCCACTAGTGGCGCGCCCGGTTGCACTTGATGAATAAG	
	SlTPL2-2YC-F	CATTTACGAACGATAGTTAATTAACATGTCTTCCTTGAGTAGGGA	SlTPL2-cYFP
	SlTPL2-2YC-R	CCACCTCCTCCACTAGTGGCGCGCCCCCTTGAAGGTGTTTCTG	
	SlTPL2-2YN-F	CATTTACGAACGATAGTTAATTAACATGTCTTCCTTGAGTAGGGA	SlTPL2-nYFP
	SlTPL2-2YN-R	CCACCTCCTCCACTAGTGGCGCGCCCCCTTGAAGGTGTTTCTG	
	SlTOE1B-N-2YC-F	CATTTACGAACGATAGTTAATTAACATGTTGGATCTGAATAATGATAGTG	SlTOE1B-N-cYFP
	SlTOE1B-N-2YC-R	CCACCTCCTCCACTAGTGGCGCGCCCACCACTACCACCATCTC	
	SlTOE1B-C-2YC-F	CATTTACGAACGATAGTTAATTAACATGGAGAATCTTGATCTGAACC	SlTOE1B-C-cYFP
	SlTOE1B-C-2YC-R	CCACCTCCTCCACTAGTGGCGCGCCCGTTGGTGGGGAGACGTAG	
	SlTOE1B-DE-2YC-F	CATTTACGAACGATAGTTAATTAACATGGATAGTGTTTTTTTCAAGG	SlTOE1B-△EAR-cYFP
	SlTOE1B-DE-2YC-R	CCACCTCCTCCACTAGTGGCGCGCCCGTTGGTGGGGAGACGTAGTTGGTT	
	2YNC-F	GAATTCTCAACACAACATATAC	nYFP
	2YN-R	CGTCGCCGTCCAGCTCGACCAG	

	2YC-R	GTGTTCTGCTGGTAGTGG	cYFP
**PULL DOWN**	NpGEX4T-GST-GCR1-F	CCGCGTGGATCCCCGGAATTCATGGAAAATGCATCTTCTTC	GST-GCR1
	NpGEX4T-GST-GCR1-R	GTCACGATGCGGCCGCTCGAGCTAAAGAGAAAGCATTGTGT	
	NpGEX4T-GST-SlTOE1B-F	CCGCGTGGATCCCCGGAATTCATGTTGGATCTGAATAATGATAGTG	GST-rSlTOE1B
	NpGEX4T-GST-SlTOE1B-R	CCGCGTGGATCCCCGGAATTCATGTTGGATCTGAATAATGATAGTG	
	pET28a-HIS-SlTOE1B-F	AGAGAACAGATTGGTGGATCCATGTTGGATCTGAATAATGA	pET28a-HIS-rSlTOE1B
	pET28a-HIS-rSlTOE1B-R	GTGGTGGTGGTGGTGCTCGAGCTAGTTGGTGGGGAGACGTA	
	NpGEX4T-GST-TPL2-F	CCGCGTGGATCCCCGGAATTCATGTCTTCCTTGAGTAGGGA	GST-TPL2
	NpGEX4T-GST-TPL2-R	GTCACGATGCGGCCGCTCGAGCTACCTTGAAGGTGTTTCTGATG	
	pET28a-HIS-GCR1-F	AGAGAACAGATTGGTGGATCCATGGAAAATGCATCTTCTTC	pET28a-HIS-GCR1 and pET28a-HIS-mGCR1
	pET28a-HIS-GCR1-R	GTGGTGGTGGTGGTGCTCGAGCTAAAGAGAAAGCATTGTGT	
	pET28a-HIS-GCR2-F	AGAGAACAGATTGGTGGATCCATGACAAGTGATTCCATTGATCTGA	pET28a-HIS-GCR2 and pET28a-HIS-mGCR2
	pET28a-HIS-GCR2-R	GTGGTGGTGGTGGTGCTCGAGTTAGGTTGCACTTGATGAATAAG	
**Y1H**	GCR1-pJG-F	CAGATTATGCCTCTCCCGAATTCATGGAAAATGCATCTTCTTC	GCR1-pJG
	GCR1-pJG-R	GAAGAAGTCCAAAGCTTCTCGAGCTAAAGAGAAAGCATTGTG	
	GCR2-pJG-F	CAGATTATGCCTCTCCCGAATTCATGACAAGTGATTCCATTG	GCR2-pJG
	GCR2-pJG-R	GAAGAAGTCCAAAGCTTCTCGAGTTAGGTTGCACTTGATGAA	
	GCR1-mMYB-pJG-F	CAGATTATGCCTCTCCCGAATTCATGGAAAATGCATCTTCTTC	GCR1mMYB-pJG
	GCR1-mMYB-pJG-R	GAAGAAGTCCAAAGCTTCTCGAGAAGAGAAAGCATTGTGTTTATATCT	
	GCR2-mMYB-pJG-F	CAGATTATGCCTCTCCCGAATTCATGACAAGTGATTCCATTG	GCR2mMYB-pJG
	GCR2-mMYB-pJG-R	GAAGAAGTCCAAAGCTTCTCGAGTTAGGTTGCACTTGATGAATAAG	
	pGCR1-1-placzi-F:	GATGAATTGAAAAGCTTGAATTCCGTGAAGATCCTGGCCGCATG	pGCR1-1-placzi
	pGCR1-1-placzi-R:	ACATACAGAGCACATGCCTCGAGGGACAATGTTCAAATCAAAACTG	
	pGCR1-2-placzi-F:	GATGAATTGAAAAGCTTGAATTCCTTTCCTCTTATACATATGTTG	pGCR1-2-placzi
	pGCR1-2-placzi-R:	ACATACAGAGCACATGCCTCGAGTTAGAAGAAGGATCAGACCAG	
	pGCR2-1-placzi-F:	GATGAATTGAAAAGCTTGAATTCGTACAATCCATAAAGAAAG	pGCR2-1-placzi
	pGCR2-1-placzi-R:	ACATACAGAGCACATGCCTCGAGCATTTTTTAAAAATTGTGTTC	
	pGCR2-2-placzi-F:	GATGAATTGAAAAGCTTGAATTCGAAATGAAGAAGAAACTTC	pGCR2-2-placzi
	pGCR2-2-placzi-R:	ACATACAGAGCACATGCCTCGAGGAGACTTTATAATATTATATTATC	
	pLFS-2-placzi-F:	GATGAATTGAAAAGCTTGAATTCGGATAAAATTACAAGTTAAC	pLFS-2-placzi
	pLFS-2-placzi-R:	ACATACAGAGCACATGCCTCGAGCTGTATTACTCTGAGGACGC	
	pLFS-4-placzi-F:	GATGAATTGAAAAGCTTGAATTCCATCACGACGCTAGATTGAC	pLFS-4-placzi
	pLFS-4-placzi-R:	ACATACAGAGCACATGCCTCGAGCGATGTCGTTGCATATATTTG	
	pLFS-5-placzi-F:	GATGAATTGAAAAGCTTGAATTCCTTTCTATTTAATGCCCGTG	pLFS-5-placzi
	pLFS-5-placzi-R:	ACATACAGAGCACATGCCTCGAGCGTTTTTGTAAATATGAA	
	Motif1-placzi-F	GATGAATTGAAAAGCTTGAATTCTAAAGAATAATAAAGAATAATAAAGAATAA	Motif1-placzi
	Motif1-placzi-R	ACATACAGAGCACATGCCTCGAGTTATTCTTTATTATTCTTTATTATTCTTTA	
	Motif2-placzi-F	GATGAATTGAAAAGCTTGAATTCATAAGAATTAATAAGAATTAATAAGAATTA	Motif2-placzi
	Motif2-placzi-R	ACATACAGAGCACATGCCTCGAGTAATTCTTATTAATTCTTATTAATTCTTAT	
	Motif3-placzi-F	GATGAATTGAAAAGCTTGAATTCAAGGGAATCTAAGGGAATCTAAGGGAATCT	Motif3-placzi
	Motif3-placzi-R	ACATACAGAGCACATGCCTCGAGAGATTCCCTTAGATTCCCTTAGATTCCCTT	
	Motif4-placzi-F	GATGAATTGAAAAGCTTGAATTCTAAATGGGAATAAATGGGAATAAATGGGAA	Motif4-placzi
	Motif4-placzi-R	ACATACAGAGCACATGCCTCGAGTTCCCATTTATTCCCATTTATTCCCATTTA	
	Motif5-placzi-F	GATGAATTGAAAAGCTTGAATTCCACATTCTTTCCACATTCTTTCCACATTCTTTC	Motif5-placzi
	Motif5-placzi-R	ACATACAGAGCACATGCCTCGAGGAAAGAATGTGGAAAGAATGTGGAA	

		AGAATGTG	
	Motif6-placzi-F	GATGAATTGAAAAGCTTGAATTCCAAATTCTATACAAATTCTATACAAATTCTATA	Motif6-placzi
	Motif6-placzi-R	ACATACAGAGCACATGCCTCGAGTATAGAATTTGTATAGAATTTGTATAGAATTTG	
	Motif7-placzi-F	GATGAATTGAAAAGCTTGAATTCTAAATTCTTCTTAAATTCTTCTTAAATTCTTCT	Motif7-placzi
	Motif7-placzi-R	ACATACAGAGCACATGCCTCGAGAGAAGAATTTAAGAAGAATTTAAGAAGAATTTA	
	Motif8-placzi-F	GATGAATTGAAAAGCTTGAATTCTACATTCTCTCTACATTCTCTCTACATTCTCTC	Motif8-placzi
	Motif8-placzi-R	ACATACAGAGCACATGCCTCGAGGAGAGAATGTAGAGAGAATGTAGAGAGAATGTA	
	Motif9-placzi-F	GATGAATTGAAAAGCTTGAATTCAATATTCTTTTAATATTCTTTTAATATTCTTTT	Motif9-placzi
	Motif9-placzi-R	ACATACAGAGCACATGCCTCGAGAAAAGAATATTAAAAGAATATTAAAAGAATATT	
	Motif10-placzi-F	GAATTGAAAAGCTTAAAAATTATTCTCTTAAAAATTATTCTCTTAAAAATTATTCTCTT	Motif10-placzi
	Motif10-placzi-R	TACAGAGCACATGCAAGAGAATAATTTTTAAGAGAATAATTTTTAAGAGAATAATTTTT	
	Motif11-placzi-F	GATGAATTGAAAAGCTTGAATTCAGGAATATAGGAATATAGGAATAT	Motif11-placzi
	Motif11-placzi-R	ACATACAGAGCACATGCCTCGAGATATTCCTATATTCCTATATTCCT	
	Motif12-placzi-F	GATGAATTGAAAAGCTTGAATTCGGAGAATTTGAGAATTTGAGAATTT	Motif12-placzi
	Motif12-placzi-R	ACATACAGAGCACATGCCTCGAGAAATTCTCAAATTCTCAAATTCTC	
	Motif13-placzi-F	GATGAATTGAAAAGCTTGAATTCTAGAATTATAGAATTATAGAATTA	Motif13-placzi
	Motif13-placzi-R	ACATACAGAGCACATGCCTCGAGTAATTCTATAATTCTATAATTCTA	
	Motif14-placzi-F	GATGAATTGAAAAGCTTGAATTCTGGAATTATGGAATTATGGAATTA	Motif14-placzi
	Motif14-placzi-R	ACATACAGAGCACATGCCTCGAGTAATTCCATAATTCCATAATTCCA	
	Motif15-placzi-F	GATGAATTGAAAAGCTTGAATTCTGGAATAATGGAATAATGGAATAA	Motif15-placzi
	Motif15-placzi-R	ACATACAGAGCACATGCCTCGAGTTATTCCATTATTCCATTATTCCA	
	Motif16-placzi-F	GATGAATTGAAAAGCTTGAATTCAGGAATAAAGGAATAAAGGAATAA	Motif16-placzi
	Motif16-placzi-R	ACATACAGAGCACATGCCTCGAGTTATTCCTTTATTCCTTTATTCCT	
	Motif17-placzi-F	GATGAATTGAAAAGCTTGAATTCAGGAATGAAGGAATGAAGGAATGA	Motif17-placzi
	Motif17-placzi-R	ACATACAGAGCACATGCCTCGAGTCATTCCTTCATTCCTTCATTCCT	
	Motif18-placzi-F	GATGAATTGAAAAGCTTGAATTCGGGAATATGGGAATATGGGAATAT	Motif18-placzi
	Motif18-placzi-R	ACATACAGAGCACATGCCTCGAGATATTCCCATATTCCCATATTCCC	
**Luciferase assay and Co-IP assay**	0800-GCR1-F	CACTATAGGGCGAATTGGGTACCGGATTATTTAGTGGCAAGTTTG	0800-pGCR1
	0800-GCR1-R	ATCGATACCGTCGACCTCGAGAATATATTCCACTGAATTATTTTCC	
	0800-GCR2-F	CACTATAGGGCGAATTGGGTACCTCCGAAAAAGAAAACTCCAAAAG	0800-pGCR2
	0800-GCR2-R	ATCGATACCGTCGACCTCGAGGATTATTATTGATGCAAGAACAAATCCTG	
	pHBT-GCR1-F:	CTCCCCTTGCTCCGTGGATCCATGGAAAATGCATCTTCTTCATCCA	pHBT-pGCR1
	pHBT-GCR1-R:	CTCGCCCTTGCTCACAGGCCTAAGAGAAAGCATTGTGTTTATATCT	
	pHBT-GCR2-F:	CTCCCCTTGCTCCGTGGATCCATGACAAGTGATTCCATTGATCTGA	pHBT-pGCR2
	pHBT-GCR2-R:	CTCGCCCTTGCTCACAGGCCTGGTTGCACTTGATGAATAAGAAGGC	
	pHBT-SlTPL2-F:	CTCCCCTTGCTCCGTGGATCCATGTCTTCCTTGAGTAGGGA	pHBT-SlTPL2
	pHBT-SlTPL2-R:	CTCGCCCTTGCTCACAGGCCTCCTTGAAGGTGTTTCTGATG	
	pHBT-mSlTOE1B-F1	CTCCCCTTGCTCCGTGGATCCATGGATAGTGTTTTTTTCAAGG	pHBT-rSlTOE1B △EAR
	pHBT-mSlTOE1B-R1	CTCCAAAGGCAGAGAAATCCAATTCTCACCACTACCACCATC	
	pHBT-mSlTOE1B-F2	GATGGTGGTAGTGGTGAGAATTGGATTTCTCTGCCTTTGGAG	
	pHBT-mSlTOE1B-R2	CTCGCCCTTGCTCACAGGCCTGTTGGTGGGGAGACGTAGTTGGTT	
	pHBT-mGCR1-F1	CTCCCCTTGCTCCGTGGATCCATGGAAAATGCATCTTCTTC	pHBT-GCR1 △EAR
	pHBT-mGCR1-R1	TTGTCATCTGAGCTTCTGCTATCAGGTAACCATTC	
	pHBT-mGCR1-F2	GAATGGTTACCTGATAGCAGAAGCTCAGATGACAA	
	pHBT-mGCR1-R2	CTCGCCCTTGCTCACAGGCCTAAGAGAAAGCATTGTGTTTA	
	pHBT-mGCR2-F1	CTCCCCTTGCTCCGTGGATCCATGACAAGTGATTCCATTG	pHBT-GCR2 △

	pHBT-mGCR2-R1	GTCTTGATTAGTTGTGCTATCAGGCAGCAAATAATC	EAR
	pHBT-mGCR2-F2	GATTATTTGCTGCCTGATAGCACAACTAATCAAGAC	
	pHBT-mGCR2-R2	TCGCCCTTGCTCACAGGCCTGGTTGCACTTGATGAATAAGAAGGCATAGTGTTTATATC	
	pHBT-GCR1mMYB-R1	GAACGATGAAGGTCTGGAGTCGAACGTAGCCTTGGCAATT	
	pHBT-GCR1mMYB-F2	AATTGCCAAGGCTACGTTCGACTCCAGACCTTCATCGTTC	
	pHBT-GCR1-mMYB-R2	CATCATTTGTAGAACCAACTTGGATGTGGCTCTTTCTTGAC	pHBT-GCR1mMYB
	pHBT-GCR1-mMYB-F3	GTCAAGAAAGAGCCACATCCAAGTTGGTTCTACAAATGATG	
	pHBT-GCR1-mMYB-R3	CTTGCTTCGATACATCTGGGAATGACTTTTTACATGAG	
	pHBT-GCR1-mMYB-F4	CTCATGTAAAAAGTCATTCCCAGATGTATCGAAGCAAG	
	pHBT-GCR2-mMYB-R1	GAGAGATGAAGTTCAGGAGTCGATCTTAGCCTTGGCAATT	
	pHBT-GCR2-mMYB-F2	AATTGCCAAGGCTAAGATCGACTCCTGAACTTCATCTCTC	
	pHBT-GCR2-mMYB-R2	CATCATTTGAAGAACCAATTTGGAAGTTGCTCTTTCTTGAC	pHBT-GCR2mMYB
	pHBT-GCR2-mMYB-F3	GTCAAGAAAGAGCAACTTCCAAATTGGTTCTTCAAATGATG	
	pHBT-GCR2-mMYB-R3	GTTTCTTGCTTCGATACATCTGGGAGTGACTTTTTACATGGG	
	pHBT-GCR2-mMYB-F4	CCCATGTAAAAAGTCACTCCCAGATGTATCGAAGCAAGAAAC	
	pHBT-GCR1-N-F	CTCCCCTTGCTCCGTGGATCCATGGAAAATGCATCTTCTTC	PHBT-GCR1-N
	pHBT-GCR1-N-R	CTCGCCCTTGCTCACAGGCCTTTGTCCAGATTCATCAAGTTTC	
	pHBT-GCR1-C-F	CTCCCCTTGCTCCGTGGATCCATGGTGTTAGGGCATGGTAAT	PHBT-GCR1-C
	pHBT-GCR1-C-R	CTCGCCCTTGCTCACAGGCCTAAGAGAAAGCATTGTG	
	pHBT-GCR2-N-F	CTCCCCTTGCTCCGTGGATCCATGACAAGTGATTCCATTG	PHBT-GCR2-N
	pHBT-GCR2-N-R	CTCGCCCTTGCTCACAGGCCTTACTTGTCCAAATTCTCCAAG	
	pHBT-GCR2-C-F	CTCCCCTTGCTCCGTGGATCCATGCTAGGCCATGCTTATAGAAC	PHBT-GCR2-C
	pHBT-GCR2-C-R	CTCGCCCTTGCTCACAGGCCTGGTTGCACTTGATGAATAAG	
	PXSN-Flag-rSlTOE1B-F	ATGATGATAAGCCAATACTTATGTTGGATCTGAATAATG	FLAG-rSlTOE1B
	PXSN-Flag-rSlTOE1B-R	GCTAGTGGATCCCCAATACTCTAGTTGGTGGGGAGACGTAG	
	PXSN-Flag-TPL2-F	ATGATGATAAGCCAATACTTATGTCTTCCTTGAGTAGGGAACTG	FLAG-SlTPL2
	PXSN-Flag-TPL2-R	GCTAGTGGATCCCCAATACTTCAGTTAGATCCTTGGCTAAGATTC	
**Quantitative RT-PCR**	GCR1-qPCR-F1	TGGGTTTGAGGAAAAAGAATGG	GCR1-GFP qRCR
	GCR1-qPCR-R1	GGACACGCTGAACTTGTGGC	
	GCR1-qPCR-F2	GTCATGGAGGAAAATAATTCAGTGG	Endogenous GCR1 qRCR
	GCR1-qPCR-R2	GCCTTGGCAATTTTGATCGA	
	GCR2-qPCR-F	ATGGAATTGCACATATAACAGCC	GCR2 qRCR
	GCR2-qPCR-R	ATATCTGATGAGTTTTTCTCCTTGC	
	GCR2-qPCR-F2	CACTATGCTCTCTCTTTCTTTGCCTTCT	GCR2-GFP qRCR
	GCR2-qPCR-R2	ACCACCCCGGTGAACAGCT	
	GCR2-qPCR-F3	TTGGGATTGAGCACAACTAATCAAG	Endogenous GCR2 qRCR
	GCR2-qPCR-R3	TCCATCCATTTGTCAGCACATCA	
	PeGCR-qPCR-F	AGTGGGGAGGAATGTAGTGAGAAAA	PeGCR qPCR
	PeGCR -qPCR-R	GTGAAGGTCAGGAGTCCAACGAA	
	NbGCR -qPCR-F	CTCCTGAACTCCATCGCTCCTT	NbGCR qPCR
	NbGCR -qPCR-R	GCTTCTCCCTTGCATTGCTTTAT	
	SlTOE1B-qPCR-F	CTCCTCCTTCCAGCCTTCACTTT	SlTOE1B qPCR
	SlTOE1B-qPCR-R	ACCCAGCGGATTCCGACAC	
	LFS-qPCR-F	GCCCAAGCCCATTAAATCTGTA	LFS qPCR
	LFS-qPCR-R	GTCTCCCATTATGTTGTCTCCTG	
	miR156B-RT	GTCGTATCCAGTGCAGGGTCCGAGGTATTCGCACTGGATACGACGTGCTC	miR156B qRCR
	miR156B-F	GCGGCGGTGACAGAAGAGAGT	
	miRNA-R	GTGCAGGGTCCGAGGTATTC	
	U6-F	TCTAACAGTGTAGTTTGTCCCTTCG	
	U6-R	TTGTGCGTGTCATCCTTGC	
	ChipQ-pGCR1-1-F	CGTGAAGATCCTGGCCGCATG	pGCR1-1 ChipQ-qRCR
	ChipQ-pGCR1-1-R	GGACAATGTTCAAATCAAAACTG	
	ChipQ-pGCR1-2-F	CTTTCCTCTTATACATATGTTG	pGCR1-2 ChipQ-qRCR
	ChipQ-pGCR1-2-R	TTAGAAGAAGGATCAGACCAG	

	ChipQ-pLFS-2-F	TCTTCGATCAAATGCGTCCTC	pLFS-2
	ChipQ-pLFS-2-R	CGGTGTATTCAAAGAAGCGTTGT	ChipQ-qRCR
	ChipQ-pLFS-5-F	GTGCAACGCGTAAATTCTTC	pLFS-5
	ChipQ-pLFS-5-R	CGAAGTAGTTTGTGCAGGAGAG	ChipQ-qRCR
