# Supplementary material for: Spatiotemporal formation of glands in plants is modulated by MYB-like transcription factors
Source: Nat Commun. 2024 Mar 15;15:2303. doi: 10.1038/s41467-024-46683-0 (PMC10943084; doi:10.1038/s41467-024-46683-0)
Supplement: Supplementary file 3 — Description of Additional Supplementary Files [file 41467_2024_46683_MOESM3_ESM.pdf]

# <u>Description of Supplementary Data</u>

**File Name:** Supplementary Data 1

**Description:** The RNA-seq data of *S. pennellii.* There are two sheets in this file:

Sheet 1 (Table S1-1): The highly expressed genes in glandular trichomes than stem episermis removal of trichomes of *S. pennellii*.

Sheet 2 (Table S1-2): The highly expressed transcription factors in glandular trichomes of *S. pennellii*, LA4024 and LA1777.

**File Name:** Supplementary Data 2

**Description:** The RNA-seq data of *cr-gcr1/2 and pMTR1:GCR1.* There are three sheets in this file:

Sheet 1 (Table S1-1): The up-regulated genes in trichomes of *cr-gcr1/2*.

Sheet 2 (Table S1-2): The down-regulated genes in trichomes of *pMTR1:GCR1*.

Sheet 3 (Table S1-3): The transcriptional factors of up-regulated in *cr-gcr1/2* and down-regulated in *pMTR1:GCR1*.
