# Supplementary material for: Spatiotemporal formation of glands in plants is modulated by MYB-like transcription factors
Source: Nat Commun. 2024 Mar 15;15:2303. doi: 10.1038/s41467-024-46683-0 (PMC10943084; doi:10.1038/s41467-024-46683-0)
Supplement: Supplementary file 6 — Reporting Summary [file 41467_2024_46683_MOESM6_ESM.pdf]

nature portfolio

Corresponding author(s): Shuang Wu

Last updated by author(s): Feb 12, 2024

Reporting Summary

Nature Portfolio wishes to improve the reproducibility of the work that we publish. This form provides structure for consistency and transparency in reporting. For further information on Nature Portfolio policies, see our Editorial Policies and the Editorial Policy Checklist.

Statistics

For all statistical analyses, confirm that the following items are present in the figure legend, table legend, main text, or Methods section.

n/a	Confirmed	
☐	☒	The exact sample size (*n*) for each experimental group/condition, given as a discrete number and unit of measurement
☐	☒	A statement on whether measurements were taken from distinct samples or whether the same sample was measured repeatedly
☐	☒	The statistical test(s) used AND whether they are one- or two-sided<br>*Only common tests should be described solely by name; describe more complex techniques in the Methods section.*
☒	☐	A description of all covariates tested
☒	☐	A description of any assumptions or corrections, such as tests of normality and adjustment for multiple comparisons
☐	☒	A full description of the statistical parameters including central tendency (e.g. means) or other basic estimates (e.g. regression coefficient) AND variation (e.g. standard deviation) or associated estimates of uncertainty (e.g. confidence intervals)
☐	☒	For null hypothesis testing, the test statistic (e.g. *F*, *t*, *r*) with confidence intervals, effect sizes, degrees of freedom and *P* value noted<br>*Give P values as exact values whenever suitable.*
☒	☐	For Bayesian analysis, information on the choice of priors and Markov chain Monte Carlo settings
☒	☐	For hierarchical and complex designs, identification of the appropriate level for tests and full reporting of outcomes
☒	☐	Estimates of effect sizes (e.g. Cohen's *d*, Pearson's *r*), indicating how they were calculated

Our web collection on statistics for biologists contains articles on many of the points above.

Software and code

Policy information about availability of computer code

Data collection	The microscope images and fluorescence intensity were collected using Leica TCS SP8X DLS software and Carl Zeiss LSM880 software. The Scanning electron microscope images were collected using TM3030Plus. The fluorescent values of LUC and REN were collected using Gen 5. Statistical analyses were performed using GraphPad Prism and Excel.
Data analysis	HISAT2 (v2.2.1); stringtie (v2.1.7); GraphPad Prism (V8.0); Venny 2.1.0 (https://bioinfogp.cnb.csic.es/tools/venny/index.html)

For manuscripts utilizing custom algorithms or software that are central to the research but not yet described in published literature, software must be made available to editors and reviewers. We strongly encourage code deposition in a community repository (e.g. GitHub). See the Nature Portfolio guidelines for submitting code & software for further information.

Data

Policy information about availability of data

All manuscripts must include a data availability statement. This statement should provide the following information, where applicable:

- Accession codes, unique identifiers, or web links for publicly available datasets
- A description of any restrictions on data availability
- For clinical datasets or third party data, please ensure that the statement adheres to our policy

The transcriptome data generated in this study have been deposited in NCBI under accession code PRJNA1074340 (Transcriptome data of trichomes of WT, cr.gcr1/2 and pMTR1:GCR1-GFP, https://www.ncbi.nlm.nih.gov/search/all/?term=PRJNA1074340) and PRJNA1074291 (transcriptome data of trichomes and stem epidermis with removed trichome of S.pennellii, https://www.ncbi.nlm.nih.gov/search/all/?term=PRJNA1074291). All data generated or analyzed in this study including figures, supplementary figures and supplementary tables are available. Source data are provided with this paper.

Research involving human participants, their data, or biological material

Policy information about studies with human participants or human data. See also policy information about sex, gender (identity/presentation), and sexual orientation and race, ethnicity and racism.

Reporting on sex and gender	N/A
Reporting on race, ethnicity, or other socially relevant groupings	N/A
Population characteristics	N/A
Recruitment	N/A
Ethics oversight	N/A

Note that full information on the approval of the study protocol must also be provided in the manuscript.

Field-specific reporting

Please select the one below that is the best fit for your research. If you are not sure, read the appropriate sections before making your selection.

☒ Life sciences ☐ Behavioural & social sciences ☐ Ecological, evolutionary & environmental sciences

For a reference copy of the document with all sections, see

Life sciences study design

All studies must disclose on these points even when the disclosure is negative.

Sample size	All of sample size used in this study meets the requirement of statistic analysis.
Data exclusions	No data were excluded from the analysis.
Replication	Numbers of replicates were stated in the figure or legend.
Randomization	Samples were always randomly allocated into experimental groups for analysis.
Blinding	The analysis was performed blind wherever possible.

Reporting for specific materials, systems and methods

We require information from authors about some types of materials, experimental systems and methods used in many studies. Here, indicate whether each material, system or method listed is relevant to your study. If you are not sure if a list item applies to your research, read the appropriate section before selecting a response.

Materials & experimental systems

n/a	Involved in the study
☐	☒ Antibodies
☒	☐ Eukaryotic cell lines
☒	☐ Palaeontology and archaeology
☒	☐ Animals and other organisms
☒	☐ Clinical data
☒	☐ Dual use research of concern
☐	☒ Plants

Methods

n/a	Involved in the study
☒	☐ ChIP-seq
☒	☐ Flow cytometry
☒	☐ MRI-based neuroimaging

Antibodies

Antibodies used

ProteinFind Anti-GFP Mouse Monoclonal Antibody(1 1000 dilution, TransGen Biotech Co., Ltd, Cat # HT801). ProteinFind Anti-Flag Antibody 1 1000 dilution, PGM050, LABLEAD Inc. Cat # F1005
Anti-His antibody (1:3000 dilution, Abmart Cat # M20001S) and anti-GST antibody (1:3000 dilution, Abmart, Cat # M20007S); Secondary antibodies (1:3000 dilution, Sigma, Cat # A5278).
Anti-green fluorescent protein (anti-GFP antibody, Sigma, Cat # G6795), 1:200 dilution for Co-IP.

Validation

All antibodies were validated by the suppliers by staining and western blot analysis.
